# Splenoses and Other Ectopic and Heterotopic Splenic Tissue: The Use of Long-Lasting Enhancement in Contrast-Enhanced Ultrasound to Avoid Unnecessary Intervention

**DOI:** 10.3390/diagnostics16081169

**Published:** 2026-04-15

**Authors:** Kathleen Möller, Siegbert Faiss, Adrian Lim, Christian Jenssen, Christoph F. Dietrich

**Affiliations:** 1 Medical Department I/Gastroenterology, SANA Hospital Lichtenberg, 10365 Berlin, Germany; k.moeller@live.de (K.M.); siegbert.faiss@sana.de (S.F.); 2 Department of Imaging, Imperial College Healthcare NHS Trust, Charing Cross Hospital Campus, London W6 8RF, UK; a.lim@imperial.ac.uk; 3 Medical Department, Hospital Maerkisch-Oderland, 15344 Strausberg, Germany; c.jenssen@khmol.de; 4 Brandenburg Institute for Clinical Ultrasound (BIKCUS), Medical University Brandenburg, 16816 Neuruppin, Germany; 5 Center of Excellence and Education, Haldenweg 11A, 3626 Thun, Switzerland

**Keywords:** CEUS, splenosis, accessory spleen, splenovisceral fusion, heterotaxy syndromes, hypervascular tumors, intrapancreatic accessory spleen

## Abstract

This narrative review describes variants of heterotopic and ectopic spleen tissue, focusing on its appearance under contrast-enhanced ultrasound (CEUS) with SonoVue (SonoVue^®^; Bracco, Milano, Italy). Typical feature of splenic tissue with SonoVue is its long-lasting enhancement. The diagnosis of these splenic variants, in the vast majority of cases, has primarily been performed with CT, MRI, spleen-specific scintigraphy, or image-guided biopsy. In this review, we analyze published cases and also include our own case examples where CEUS has been used, and describe the enhancement characteristics of splenosis and atypical (intrapancreatic) accessory spleens. CEUS can provide valuable diagnostic information in patients with suspected ectopic splenic tissue, particularly when interpreted together with clinical history and complementary imaging modalities. Ultimately, ectopic splenic tissue should be considered, especially after splenectomy or splenic trauma, in cases of well-defined, hypervascularized lesions where CEUS may help avoid unnecessary invasive procedures in selected cases.

## 1. Introduction

Accessory spleens are common and easy to diagnose when located in typical positions within the splenic bed. The diagnosis, however, can be challenging in cases of atypical or intrapancreatic splenic tissue [1].

Splenoses refers to the autotransplantation of scattered splenic tissue as a result of spleen injury during abdominal trauma, abdominal surgery, or splenectomy. Splenoses are well vascularized and, if diagnosed years after splenectomy, can mimic well-vascularized tumors especially if embedded within an organ. In case reports and case series, CT, MRI, and spleen-specific scintigraphy have predominantly been used for characterization and it is noteworthy that surgical resection was ultimately performed in the majority of these patients. A large number of patients also underwent image-guided biopsy.

After intravenous administration of the contrast agent (SonoVue, Bracco, Milan, Italy) under contrast-enhanced ultrasound (CEUS), splenic tissue exhibits a long-lasting enhancement of up to 5 min owing to its uptake by the reticuloendothelial system. This characteristic feature allows the identification of splenic tissue, including heterotopic and ectopic spleens [2,3]. Although these enhancement characteristics are highly specific and can thus significantly influence patient outcomes, the number of publications on splenosis and accessory spleen in CEUS with SonoVue is limited. The aim of this review is to discuss the characteristics of long-lasting enhancement of splenic tissue in CEUS as a supportive diagnostic feature that may help guide differential diagnosis in conjunction with clinical history and complementary imaging modalities.

## 2. Methodology

### 2.1. Search Strategy

**Figure 1:**
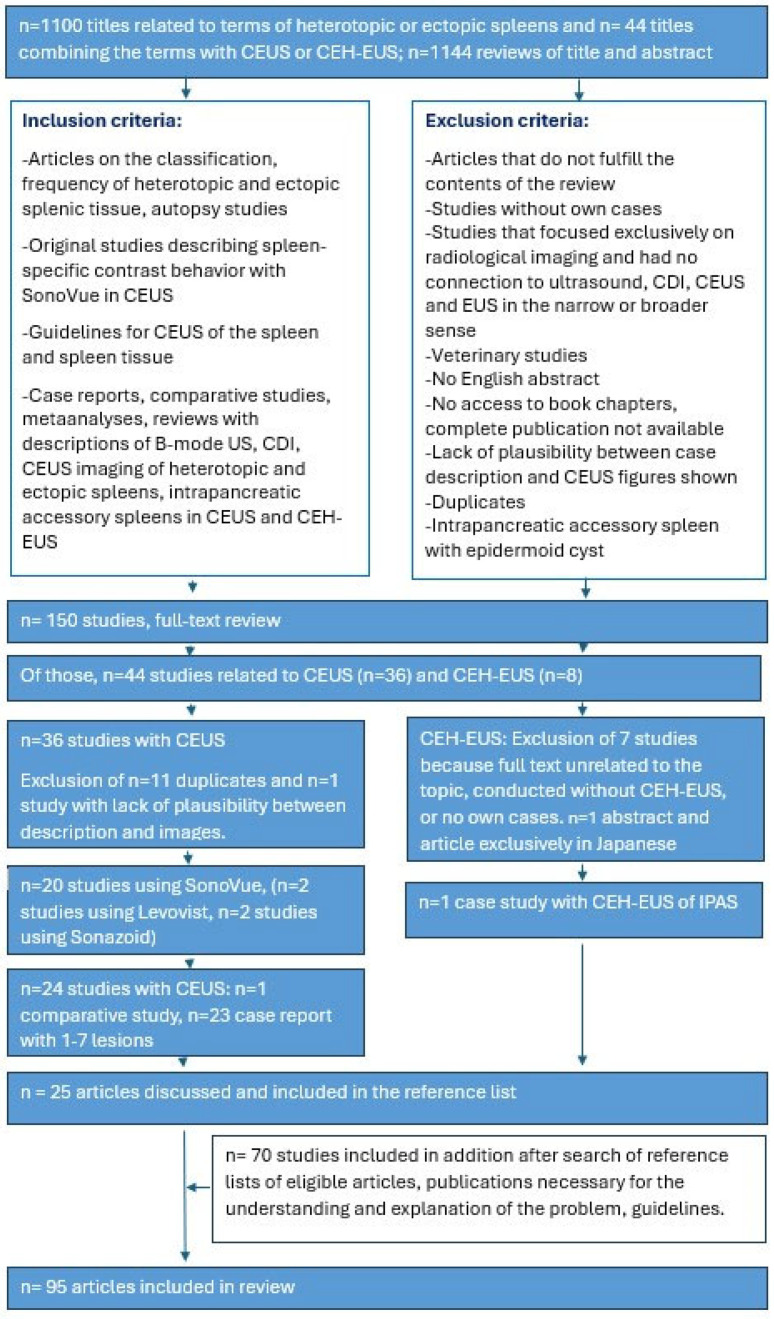
Search strategy.

A narrative review with a systematic literature search was conducted using PubMed. The search covered the period from January 1995 to 30 September 2025. The ultrasound contrast agent SonoVue (SonoVue, Bracco, Milan, Italy) has been approved since March 2001, so studies on SonoVue were only to be expected after this date. General data on the prevalence of ectopic and hereditary spleen tissue, autopsy studies, reviews of previous diagnostics in radiological methods, and specifically the appearance in CEUS and contrast-enhanced low-MI-EUS (CE-LMI-EUS) were researched. A double independent review procedure was performed (K.M., C.J.). The following keywords were used (the results are shown in parentheses): Splenosis AND Ultrasound (381); Splenosis AND Color Doppler Imaging (6); Splenosis AND CEUS (10); Ectopic spleen AND CEUS (4); Heterotopic spleen AND CEUS (1); Accessory spleen AND CEUS (6); Intrahepatic splenosis (68); Intrahepatic splenosis AND CEUS (2); Hepatic splenosis AND CEUS (3); Pancreatic splenosis (49); Pancreatic splenosis AND CEUS (1); Intrapancreatic accessory spleen (IPAS) (252); IPAS AND Color Doppler Imaging (11); IPAS AND CEUS (3); IPAS AND EUS (27); IPAS AND CE-EUS (8); Peritoneal splenosis (113); Peritoneal splenosis AND CEUS (1); Pelvis splenosis AND CEUS (1); Gastrointestinal splenosis (48); Gastrointestinal splenosis AND CEUS (0); Thoracis splenosis (2); Thoracis splenosis AND CEUS (1); Thoracal splenosis (142); Thoracal splenosis AND CEUS (1); Congenital asplenia AND CEUS (0); Polysplenia AND CEUS (0); Splenovisceral fusion (1); Splenovisceral fusion AND CEUS (0); Splenohepatic fusion AND CEUS (0); Splenopancreatic fusion AND CEUS (0); Splenorenal fusion AND CEUS (0); and Splenogonadal fusion AND CEUS (2). The literature search was performed in English. Relevant articles in other languages were read using translation systems. Articles without available abstracts were excluded unless the full publication was available. Veterinary studies, unpublished articles, or conference abstracts without methodological details were excluded. The inclusion and exclusion criteria are listed in Figure 1.

Four studies on alternative diagnostic methods were removed during the peer-review process.

Although the narrative review primarily researched enhancement with SonoVue, two case reports with Levovist (Schering AG, Berlin, Germany) and two case reports with Sonazoid (GE Healthcare AS, Oslo, Norway; Daiichi-Sankyo) have been included.

The description of the enhancement with CEUS using SonoVue was reviewed and compared with the published images. The focus was on describing the enhancement of lesions in the arterial phase and in the parenchymal phase, with particular attention to the duration of enhancement in the parenchymal phase. The reliability of the diagnosis was verified where in all case reports, this was confirmed histologically by ultrasound-guided sampling, surgery, and, in one case, EUS-guided sampling.

As the available evidence consists predominantly of case reports and small case series, this work should be interpreted as an evidence-based narrative review rather than a systematic diagnostic accuracy analysis.

### 2.2. Limitations

A key disadvantage of our study is that 24/25 studies involving CEUS and CE-LMI-EUS are case reports. Only one study on peritoneal splenosis is a comparative study. Twenty of the case reports describe only a single case. There are no multicenter studies, meta-analyses, or information on interobserver variability.

One particular challenge of our research includes the fact that the precise timing of enhancement was not available in all of the reported CEUS studies. We reviewed the images in all publications for comparison. However, the exact time of enhancement could usually not be determined.

## 3. Heterotopic and Ectopic Spleen Tissue

The spleen is located subphrenically and intraperitoneally in the upper-left abdomen. In addition, there are heterotopic and ectopic splenic conditions [4,5]. Heterotopic spleen arises from incomplete fusion of splenic tissue (accessory spleen) or from abnormal splenovisceral fusion. Ectopic conditions include splenosis, wandering spleen, polysplenia, and asplenia. These occur post-traumatically or post-operatively as splenosis or as a wandering spleen resulting from weakness or malformation of the gastrosplenic, phrenicosplenic and splenorenal ligaments. Polysplenia and asplenia are seen in the context of heterotaxy syndromes [4,5] [Table 1]. Ultimately, both are splenic tissue in a different location.

### 3.1. Accessory Spleen

Accessory spleen is a congenital condition, the result of failed fusion of splenic tissue during embryonic development along the path from where the spleen forms in the midline to the spleen’s final location within the left side of the abdomen [6,7,8]. Accessory spleens are usually asymptomatic incidental findings [1]. Approximately 10–20% of adults have accessory splenic tissue. In the majority of cases, there is only one accessory spleen, but in 8–20% of cases, two or three accessory spleens have been found. Although 80% of accessory spleens are located within the splenic hilum, near the lower or adjacent to the tail of the pancreas [8,9,10,11,12,13], they may also be found anywhere along the splenic vessels, in the gastrosplenic ligament, the splenorenal ligament, the walls of the stomach or intestines, the pancreatic tail, the greater omentum, the mesentery, or the gonads and along their path of embryological descent [14]. Very rare manifestations of the retroperitoneal or gonadal localization are not included in the current accessory spleen classification in the “Terminologia Embryologica” [4].

In a study of 3000 autopsies, Halpert and Györkey [11] report on 311 patients (10.5%) in whom 364 accessory spleens were found. Of these 364 accessory spleens, 61 (16.7%) were located intrapancreatically. Of these, 59 intrapancreatic accessory spleens were solitary and one patient had two intrapancreatic accessory spleens. The frequency of an intrapancreatic accessory spleen was 2% of all patients and a double intrapancreatic accessory spleen was found in 0.07%. All intrapancreatic accessory spleens (100%) were located in the pancreatic tail [11].

Most accessory spleens are round; less commonly, they are oval or triangular. The echotexture is identical to that of the splenic parenchyma.

They are well-encapsulated and contain a normal vascular hilum within normal spleen tissue and are supplied by small branches of the splenic artery. Accessory spleens usually have a central or eccentric but symmetrical vascular tree [15].

**Figure 2:**
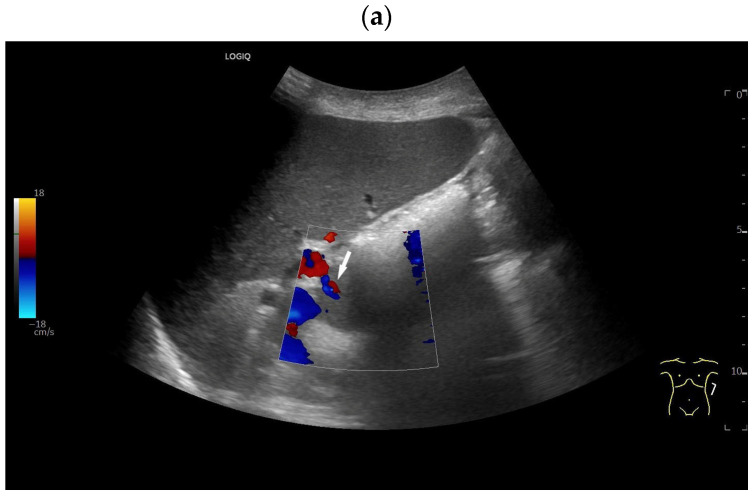

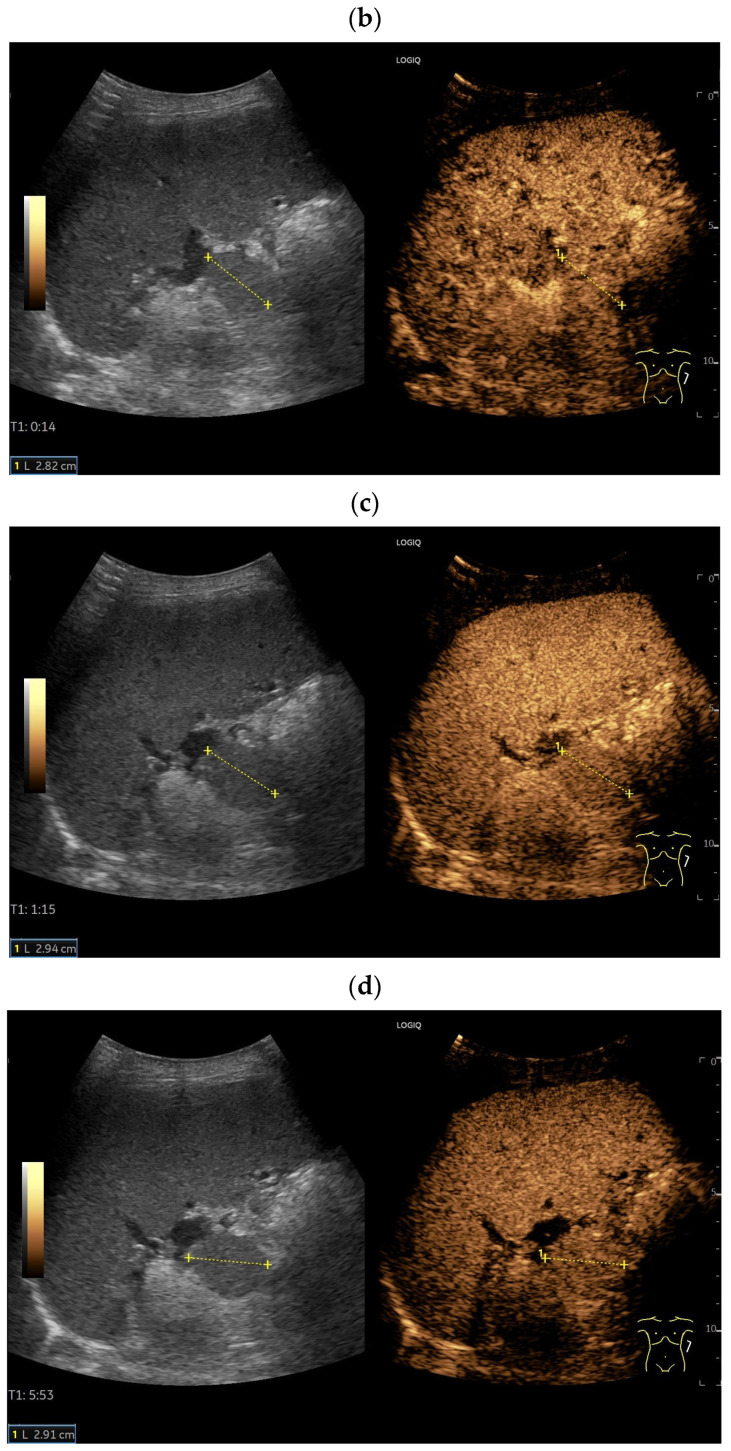
Accessory spleen. Incidental finding of a 28 mm smooth-bordered lesion at the splenic hilum with similar echogenicity to the splenic parenchyma. Visualization of two small afferent and efferent vessels (arrow), apparently supplied by the adjacent splenic artery (**a**). Owing to its proximity to the pancreatic tail and its size, a pancreatic tail tumor needed to be excluded. During the same initial US examination, CEUS was performed using 1.2 mL of SonoVue. Like the spleen, the lesion (between the markers) showed heterogeneous enhancement in the arterial phase (14 s) (**b**), followed by homogeneous enhancement (**c**). The examination continued for 6 min (**d**) where the lesion showed enhancement identical to that of the adjacent spleen. The diagnosis of a spenunculus was made, and a hypervascularized pancreatic tumor was excluded, requiring no further diagnostic tests.

In the splenic hilum, they can mimic pathological lymph nodes or tumors of the pancreatic tail; in other locations, they can mimic well-vascularized tumors; and in the pancreatic tail, it is important to differentiate them from neuroendocrine tumors and metastases from renal cell carcinomas [Figure 2].

### 3.2. Splenoses

Splenoses describes ectopic splenic tissue secondary to autotransplantation after splenic trauma and/or splenectomy. In most locations, splenoses are asymptomatic incidental findings whose diagnostic significance lies in distinguishing them from well-vascularized tumors of the corresponding organ.

The first case of splenosis was described by Albrecht in 1896, and the condition was named by Buchbinder and Lipkoff in 1939 [16,17,18]. Splenosis occurs in up to 67% of patients after traumatic splenic rupture and splenectomy [19].

Scattered fragments of spleen tissue interact with the surrounding fibrous tissue and form a matrix that subsequently supports differentiation into lymphocytes, endothelial sinusoids, capillaries, and ultimately spleen-like tissue, resulting in autologous ectopic implantation.

There are several mechanisms of splenic tissue dissemination [20]. The most common is the direct implantation of spleen fragments after trauma on adjacent organs and structures (e.g., omentum, parietal peritoneum, or the serosal surface of the intestinal wall). If there is a simultaneous diaphragmatic injury, fragments of the spleen may enter the thoracic cavity. Furthermore, intraoperative splenic fragments can be implanted in distal peritoneal surfaces through peritoneal lavage. Last but not least, hematogenous seeding can occur via splanchnic circulation to distant organs, such as the liver.

Typical locations are the serosal surface of the small bowel, greater omentum, parietal peritoneum and serosal surface of the large intestine and mesentery. Less common locations are intrahepatic, intrapancreatic, renal and intrathoracic implants. The high regenerative capacity of splenic tissue has been used, for example, in autotransplantation to partially restore the immunological function of the spleen after splenectomy [21].

In general, one must distinguish between accessory spleens and splenosis [Table 2]. Splenoses are unencapsulated or poorly encapsulated, have no characteristic shape and contain some distorted spleen tissue. Spleen tissue in splenosis usually reveals modified architecture. Normal splenic vasculature is absent in the implanted spleen tissue, which may result in different blood supply. Splenoses have no vascular hilum and receive their blood supply from surrounding tissues [6,22,23,24]. The histological descriptions of splenosis vary. Some authors state that splenosis differs from a normal spleen at the microscopic level. The red pulp is generally described as normal compared to other spleen tissue, but there are differing reports regarding the white pulp. The white pulp can be either normal or poorly developed [25,26]. Carr and Turk [25] describe examples in which splenoses were histologically indistinguishable from normal splenic tissue. They described a well-developed white pulp with central arterioles, focal germinal centers, and the expected distribution of T and B lymphocytes.

### 3.3. Peritoneal and Pelvic Splenoses

The peritoneal cavity is the most common location for scattered spleen implants [27,28]. Manifestation in the pelvis is less common. However, splenoses can attach themselves to the adnexa or are localized on the serosal surface of the intestinal loops [29,30,31].

### 3.4. Intrahepatic Splenosis

Intrahepatic splenoses are very rare. They are more frequently located on the left than in the right liver lobe and are usually subcapsular [16,32]. The development of hepatic splenosis is explained by hematogenous seeding of splenic fragments onto serosal surfaces during splenic trauma or splenectomy and cell proliferation, promoted by local hypoxia of the liver [16].

The published cases of intrahepatic splenosis since 1993 have been evaluated in several reviews [33,34,35]. The majority were single intrahepatic splenoses; 6.8% of the cases included both intrahepatic and extrahepatic splenoses [35]. Up to 40% of all cases had hepatitis B infection, 55% had hepatitis C and 55% had liver cirrhosis [33]. And 94.6% of patients with intrahepatic splenosis had a history of trauma and/or splenectomy [33]. The majority were men (83%), more likely due to trauma and an apparently more dangerous lifestyle. The median time from splenectomy to diagnosis of intrahepatic splenosis was 21 years (range 1.5–47 years) [34]. Sato et al. express the hypothesis that erythropoiesis induced by local hypoxia of the liver with progression of chronic hepatitis C may trigger the rapid growth of splenic implants [23].

Usually, intrahepatic splenoses are asymptomatic incidental findings. Their significance lies in the diagnostic differentiation from other liver tumors, especially in patients with pre-existing liver disease. In a case report and literature analysis of all cases up to 2018, intrahepatic splenosis was considered as the primary diagnosis in only 15.5% of cases. HCC was considered the primary diagnosis in 49.2% of cases. Surgical intervention was performed in a total of 63% of patients because the diagnosis of intrahepatic splenosis could not be confirmed by imaging (CT, MRI, or Scintigraphy) or US-guided needle biopsy [34]. In a case report and literature review with evaluation of all 80 published cases of intrahepatic splenoses from 1993 to 2025, the histological diagnosis was confirmed in 57% of cases by surgical resection and in 21% by biopsy [35]. In patients who underwent surgery, the preoperative suspected diagnosis was HCC in 48%, while in others it was suspected hepatocellular adenoma or liver metastasis [35].

### 3.5. Thoracic Splenosis

Intrathoracic splenosis tends to occur on the left intrathoracic side and is associated with traumatic diaphragmatic injury [36,37,38]. The lesions are usually peripheral, round or oval, and smoothly defined. They may occur in chains. Intrathoracic splenoses receive their blood supply from the pleura, the chest wall, or the diaphragm [37]. Thoracic splenosis is usually asymptomatic; however, in rare cases (<10–15% of cases), patients can exhibit clinical symptoms such as chest pain, hemoptysis, or coughing. These symptoms are attributed to mechanical irritation and pressure caused by the lesion [36].

Thoracic splenoses can only be diagnosed by US and CEUS if they are located on the chest wall and are not obscured by lung [28,39].

### 3.6. Gastrointestinal Splenosis

By 2024, 24 gastric splenosis case reports had been published [26]. Gastric splenosis was mostly located in the fundus, but also in all other regions of the stomach. They appeared as spherical impressions with intact mucosa. The masses were predominantly round or oval, with only a few having blurred or polycyclic boundaries. They were hypoechoic on ultrasound, may have calcifications, and the predominantly suspected diagnosis was of a gastrointestinal stromal tumor [26]. A hemangioma was described in one of the gastric splenoses [40]. The majority of patients had upper abdominal discomfort or dyspeptic symptoms, which led to diagnostic procedures. However, these could not be clearly attributed to splenosis.

Seventeen cases of splenosis in the small intestine and colon had been published up to 2021 [41]. The majority of patients had obstructive symptoms with abdominal pain.

We were unsuccessful in our search for descriptions of the use of CEUS or CE-LMI-EUS in gastrointestinal splenoses.

### 3.7. Splenovisceral Fusion

Splenovisceral fusion is a very rarely observed congenital result of migration and fusion of splenic tissue with developing visceral organs during embryogenesis [Table 3]. Splenogonadal, splenohepatic, splenorenal, splenopancreatic and splenoadrenal fusions have been described [5,42,43,44,45]. Splenovisceral fusion is more frequently, but not exclusively, located on the left side [43]. In splenogonadal fusion, a distinction is made between complete and incomplete variants. This depends on whether there is a connecting fibrous band between the left gonad and the spleen, with or without associated splenic tissue along its path [5,46].

Splenopancreatic fusion is described either as ectopic spleen tissue in the cauda pancreatis, or as ectopic pancreatic tissue in the spleen or accessory spleen, or as fusion of the cauda pancreatis and splenic hilum [47]. The intrapancreatic accessory spleen is likely the most common normal variant. Splenopancreatic fusion can be associated with trisomy 13 [4,47].

**Figure 3:**
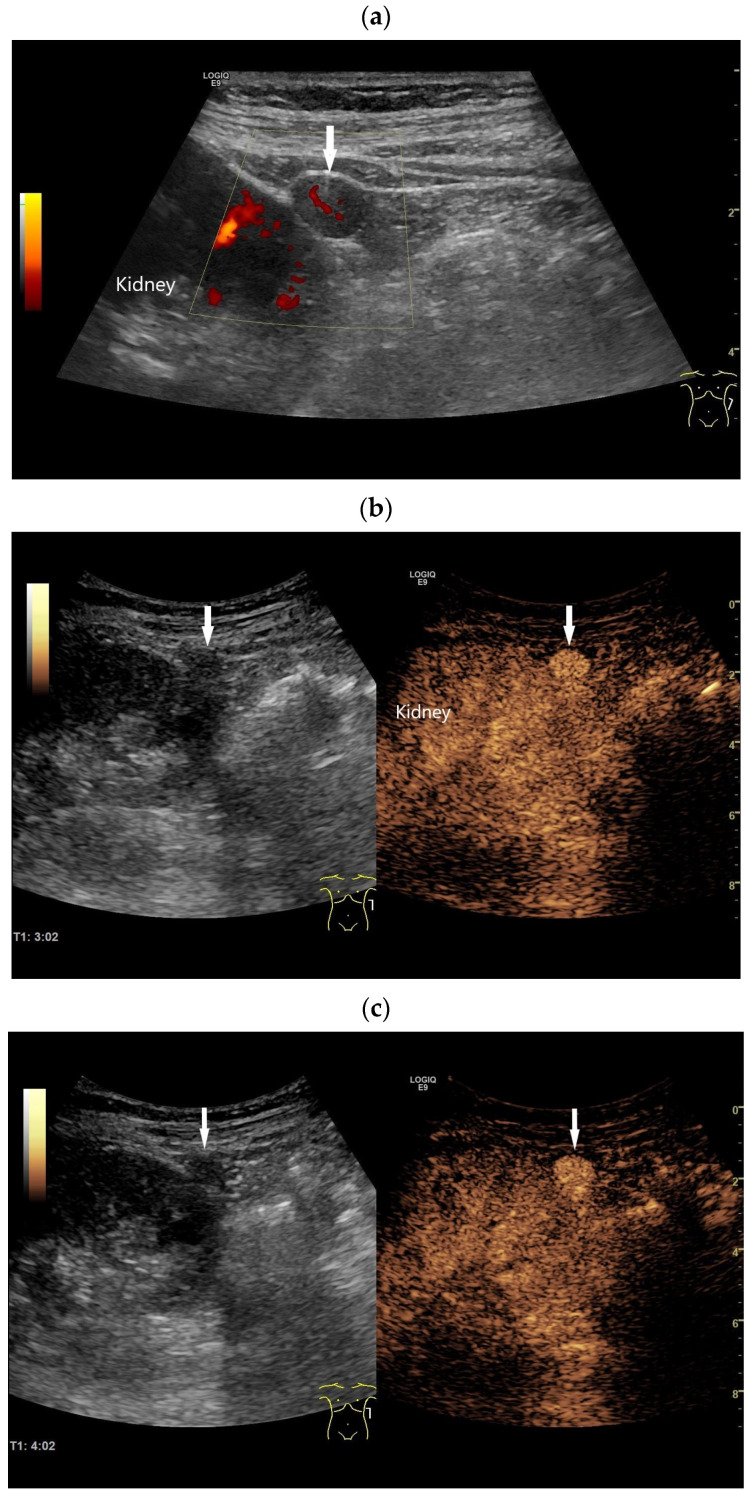

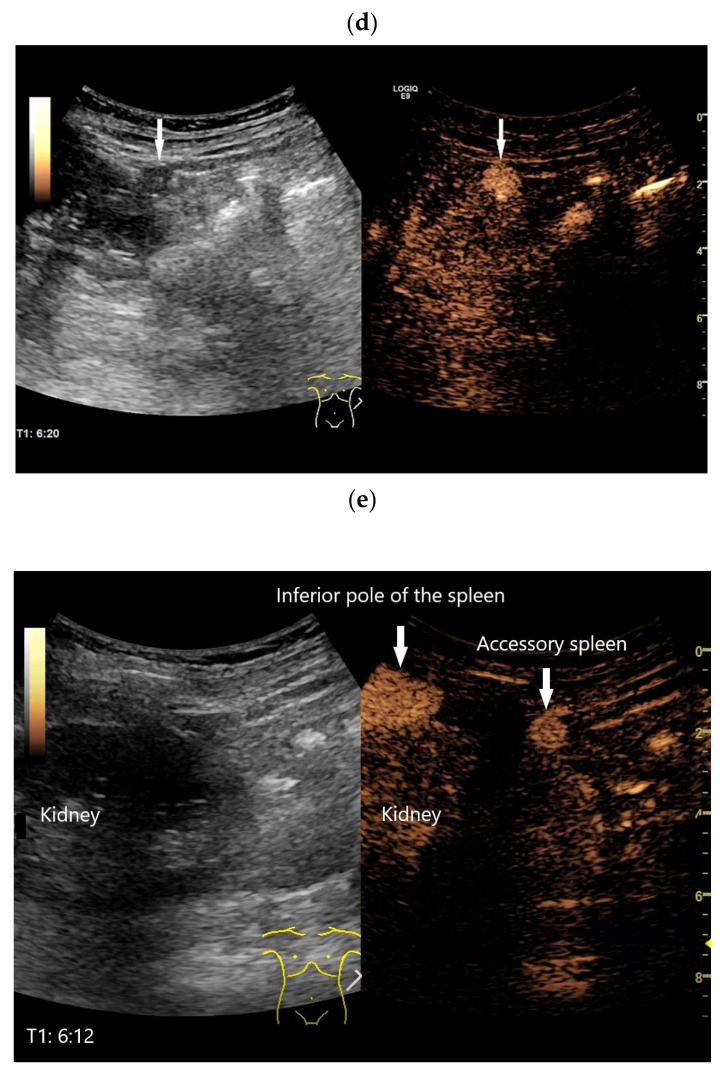
Accessory spleen on the left kidney/splenorenal fusion. Incidental finding of a 9 mm smooth-bordered, homogeneous lesion (arrow) with a central vessel on the surface of the left kidney (**a**). Suspected diagnosis of a solid renal tumor until proven otherwise. CEUS with 1.2 mL of SonoVue showed a hyperenhanced lesion in all phases. From the 3rd minute (**b**) onward, the enhancement of the renal parenchyma decreased; however, the lesion (arrow) was hyperenhanced. Further imaging was performed at intervals of one minute until 6:20 min. While there was little visible enhancement in the surrounding tissue, the lesion (arrow) remained unchanged and hyperenhanced (**c**,**d**). The enhancement of the spleen (arrow) and the accessory spleen (arrow) are identical after more than 6 min (**e**).

The differential diagnostic significance lies in distinguishing heterogeneous splenic tissue from well-vascularized solid tumors of the affected organs [Table 3] [Figure 3]. Palpable textural change in the testes may mimic tumors.

### 3.8. “Wandering” Spleen

This is a variant in which the otherwise morphologically unremarkable spleen is located dystopically in the abdomen or pelvis. The dystopic position is caused by the weakness, absence, or malformation of the normal spleen ligaments [5,48]. On imaging, the spleen is missing from its normal subphrenic location in the left upper abdomen. The spleen can then often be found in a different non-specific location and this is only of clinical significance when there is the possibility of vascular and splenic torsion with splenic infarction and presentation of an acute abdomen [49,50]. Intestinal obstruction caused by a wandering spleen has also been described [51]. A high degree of clinical experience is required to interpret the situation correctly and CEUS is indicated if splenic infarct is suspected [3]. However, there are no case reports of infarction of a “wandering spleen” diagnosed with CEUS.

### 3.9. Heterotaxy Syndromes (Asplenia and Polysplenia)

**Figure 4:**
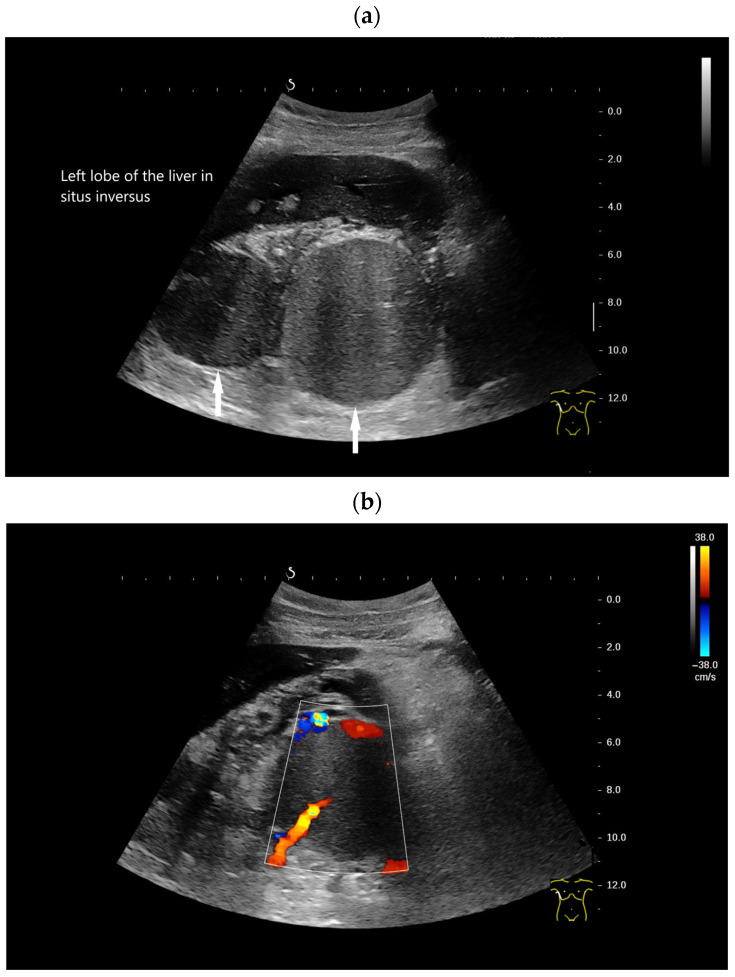

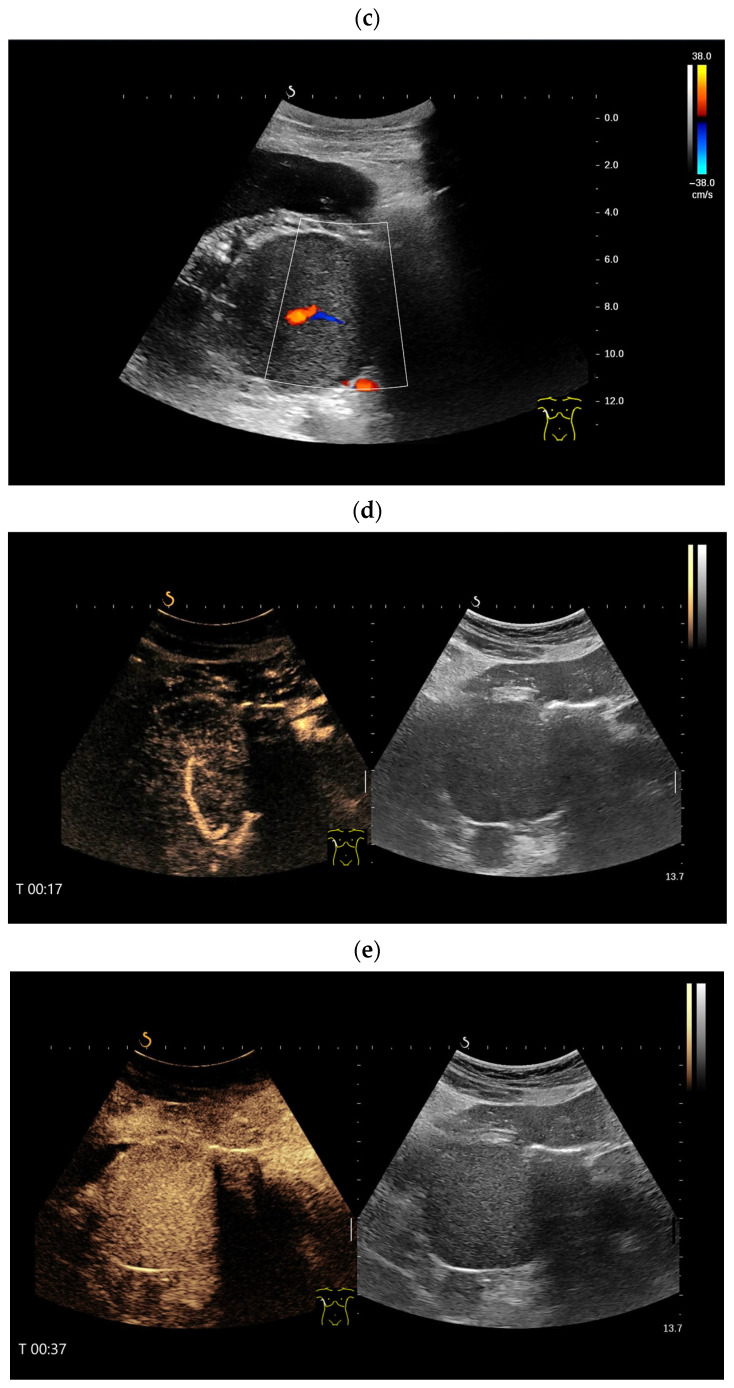

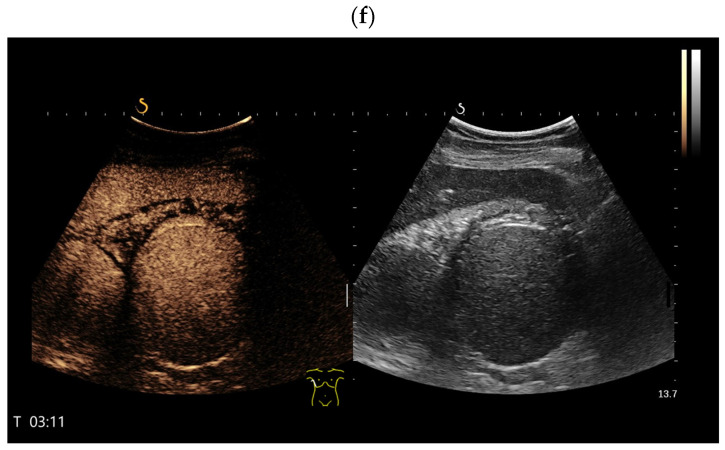
Kartagener syndrome (immobility of cilia in the airways and recurrent infections of the respiratory tract, situs inversus, bronchiectases, sinusitis). History of traumatic splenectomy following a motor vehicle accident. The transducer is positioned in the right 10th–11th intercostal space, demonstrating the liver extending far to the right due to situs inversus. Under normal anatomical conditions, the hepatic hilum would be visualized at this location. Distal to the transducer, two larger round structures (arrows) measuring up to 8 cm in diameter are identified (**a**). CDI and CEUS reveal a central feeding vessel with a branching vascular tree (**b**–**d**) as well as sustained contrast enhancement over several minutes (**e**,**f**), findings typical of accessory spleens. On B-mode imaging, several additional adjacent structures cannot be reliably delineated; however, contrast-enhanced ultrasound clearly demonstrates these lesions as further splenunculi. In the context of situs inversus and Kartagener syndrome, if splenic structures are located on the right side, and several large structures are detected, polysplenia should be considered as the differential diagnosis rather than splenosis.

Heterotaxy syndromes comprise a spectrum of abnormal disorders affecting the typical asymmetrical positions of the visceral organs in the chest and abdomen along the right and left axes [5,52,53]. Other significant congenital anomalies are common in these patients. The two main subtypes are asplenia and polysplenia, with varying degrees of organ displacement [5]. While polysplenia can cause many small spleens to develop along the greater curvature of the stomach, asplenia involves the absence of the spleen. Both congenital anomalies are associated with other congenital anomalies, most of which are severe [5,52,53] [Figure 4].

## 4. Imaging

### 4.1. Spleen Tissue on CEUS

The spleen consists of two different types of tissue: the hematopoietic red pulp, which removes damaged erythrocytes and other cell debris, and the immunological white pulp, which activates the immune system’s response [5,25]. Red and white pulp have different blood flow rates. This is important for the appearance on various contrast-enhanced imaging techniques.

In most organs with an arterial blood supply, except the liver and lungs, two vascular phases are described. The arterial phase begins approximately 10–20 s after contrast agent administration and lasts until approximately 35–40 s. The venous or parenchymal phase begins in these organs approximately 30 to 45 s after contrast agent administration. In the spleen, the arterial (10–35 s) and late parenchymal phases (3–5 min) are the most valuable diagnostically.

During the arterial phase in CEUS, up to 30 s post injection, the spleen exhibits a unique heterogeneous enhancement known as the “zebra effect” [3]. The cause of this is believed to be the different flow of blood through the two circulatory systems within the spleen, the red and white pulp. The same “zebra” appearance has also been described in the arterial phase of contrast-enhanced CT and MRI examinations of the spleen. The inhomogeneous enhancement on CEUS lasts for approximately 30 s and then becomes homogeneously hyper-enhancing up to 60 s after injection. This homogeneous hyperenhancement persists for at least 5 min. [54,55]. The contrast behavior of splenic tissue with the ultrasound contrast agent (UCA) SonoVue (SonoVue^®^; Bracco, Milano, Italy), compared to the liver and the kidneys, was investigated by Lim et al. [2]. While the liver and both kidneys show a decrease in total uptake over 5 min, the total uptake of the spleen remained constant at 99%. Further observations described an even longer enhancement of spleen tissue [27,28,29,54]. Lim et al. concluded from their results that CEUS with SonoVue is well suited for the diagnosis of splenic tissue owing to its long-lasting enhancement [2]. The EFSUMB guidelines of nonhepatic CEUS consider late parenchymal enhancement as a differential diagnostic possibility to distinguish accessory spleen and splenosis from pathological masses [3]. These recommendations highlight the diagnostic relevance of persistent parenchymal enhancement patterns for identifying splenic tissue in atypical locations.

### 4.2. Accessory Spleen and Splenosis in Ultrasound, Color Doppler Imaging and CEUS

**Figure 5:**
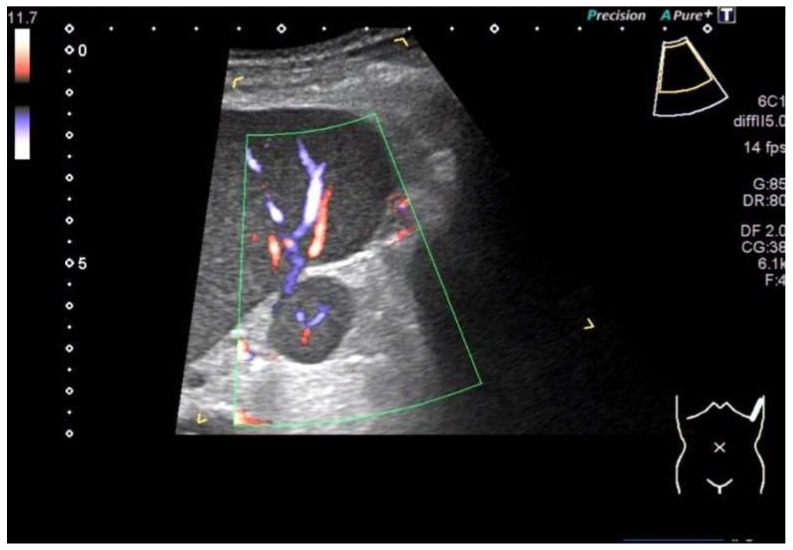
A typical case of an accessory spleen located between the hilum and the lower pole of the spleen. B-Mode ultrasound shows a roundish solid focal lesion of 2 × 1.8 cm with a smooth outline, which has the same echogenicity and echopattern as the spleen. Color Doppler ultrasound shows a centrally branching accessory splenic artery and intrasplenic arterial and venous vessels.

On B-Mode ultrasound, accessory spleens appear round, with smooth borders and exhibit the same parenchymal echogenicity as the spleen [56]. On color Doppler imaging (CDI), a feeding vessel can occasionally be seen, and sometimes also the intrasplenic vascular branches [56] [Figure 5]. Case reports on intrahepatic splenosis describe round or oval shapes and lobulated margins [56], a heterogeneous hypoechoic lesion with a hyperechoic [23] or hypoechoic rim [57], a slightly more echogenic lesion compared to the surrounding parenchyma [58], or a homogeneous, round lesion with demarked margins [24]. Peripheral vessels indicate the blood supply of splenosis from surrounding tissue [56]. In a case of intrahepatic splenosis, CDI demonstrated a “dotted” and “striped” appearing blood flow signal internally and at the border of the lesions [24].

Despite their different architecture, splenoses and accessory spleens have the same behavior in CEUS as the normal spleen [3,6]. But direct comparison of the enhancement is not possible if the spleen is removed. The enhancement usually persists for more than 5 min post injection [3,55]. In clinical practice, CEUS findings should always be interpreted in conjunction with the clinical history and complementary imaging modalities such as MRI or CT. In diagnostically uncertain cases, image-guided biopsy or EUS-guided sampling may be required for definitive confirmation.

The following sections summarize organ-specific manifestations of ectopic and heterotopic splenic tissue and the corresponding imaging findings.

### 4.3. Peritoneal

**Figure 6:**
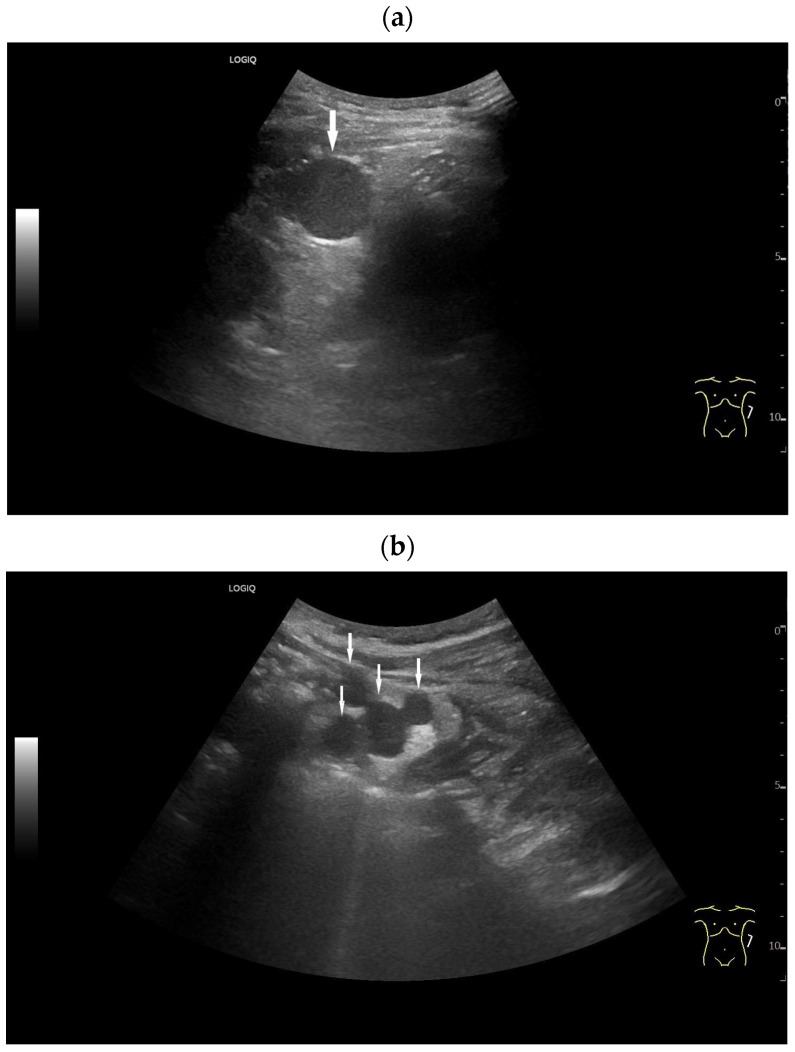

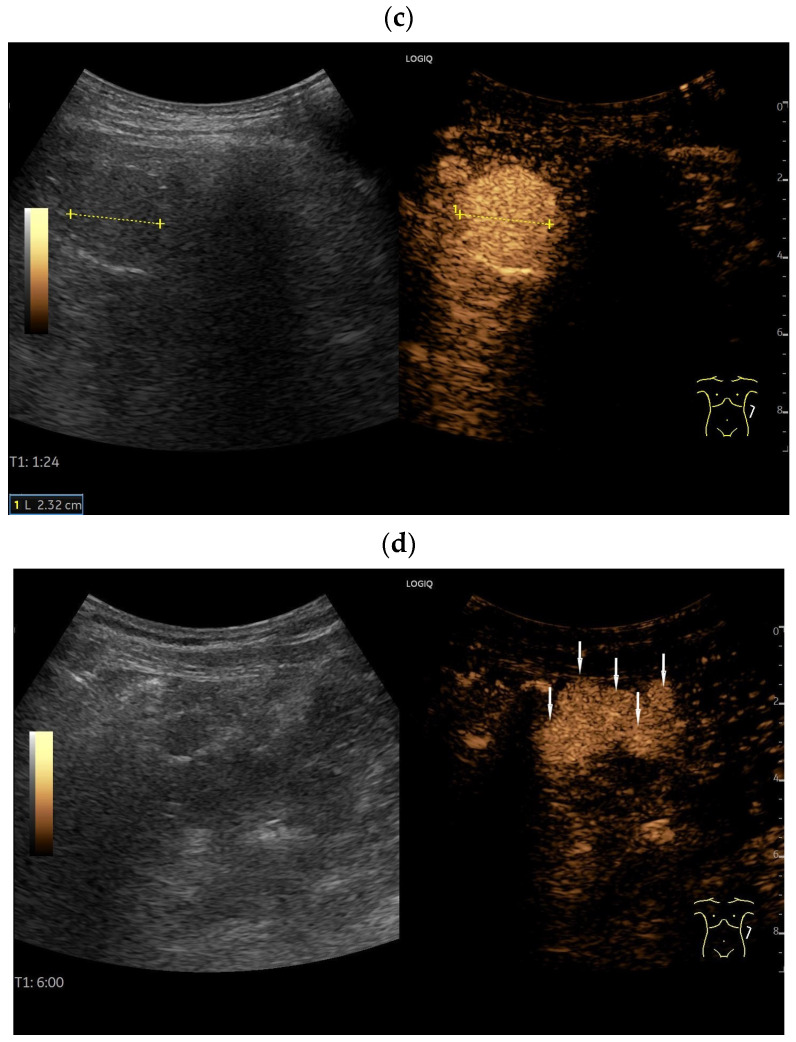
Splenosis. Incidental finding of several round, homogeneous lesions in a patient with a history of post-traumatic splenectomy. The largest lesion, measuring 25 mm (arrow) (**a**), was adjacent to the left kidney. At least four smaller lesions (arrows), up to 15 mm in diameter, were located interenteric (**b**) and suggested mesenteric lymphadenopathy. Contrast-enhanced ultrasound (CEUS) using 1.2 mL of SonoVue showed hyperenhancement in all lesions until the end of the examination at 6 min. The larger lesion (between the markers) is visualized after 3 min (**c**), and the smaller ones (arrows) after 6 min (**d**). Splenosis was diagnosed in conjunction with the previous splenectomy. No further investigation was necessary.

**Figure 7:**
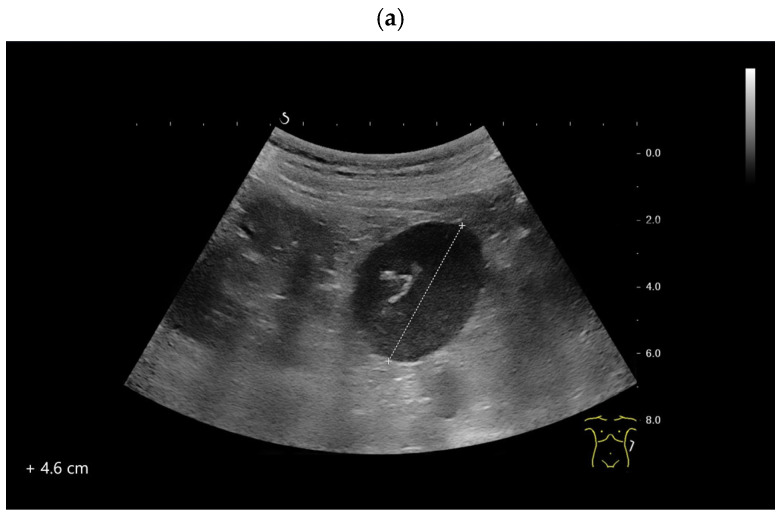

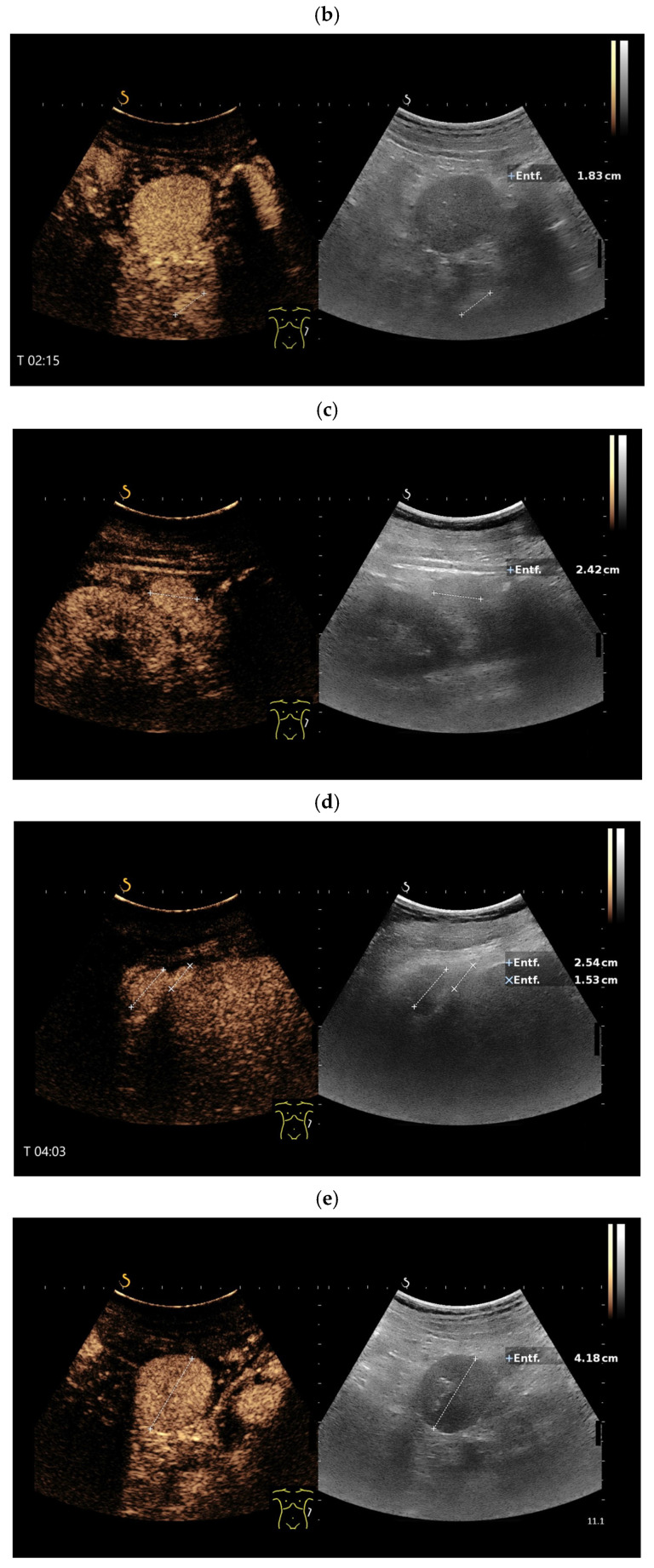
Splenosis. In a patient with a history of splenectomy, a 46 mm smooth-bordered hypoechoic lesion (between the markers) with echogenic internal reflections is visible in the left upper abdomen (**a**). On CEUS, the lesion shows a homogeneous and prolonged enhancement (**b**). Additionally, further contrast-enhancing lesions (between the calipers) are also visible, which were previously not discernible in B-mode ultrasound (**c**–**e**). Hyperenhancement was visible for longer than 5 min. The figure demonstrates the prolonged enhancement of the lesions; here at 4:23 min (**e**). Entf. = distance.

**Figure 8:**
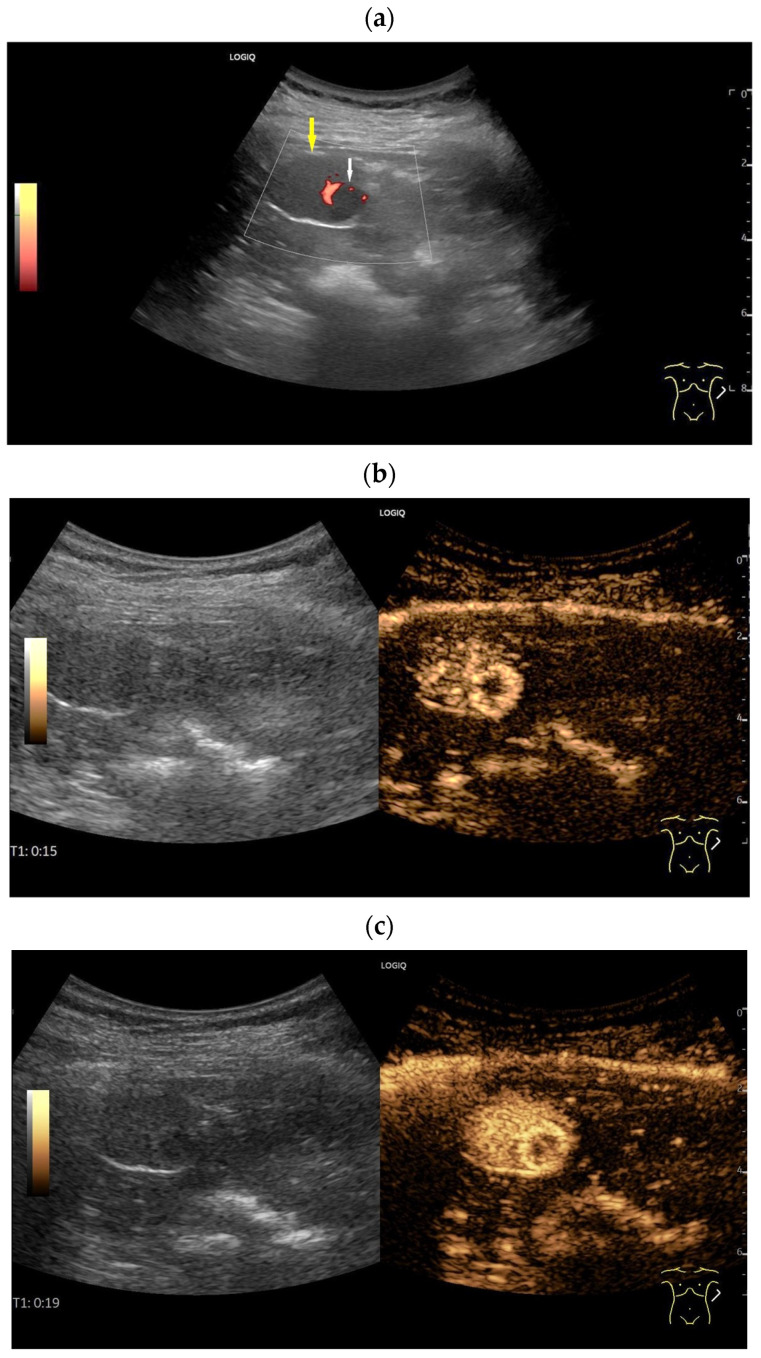

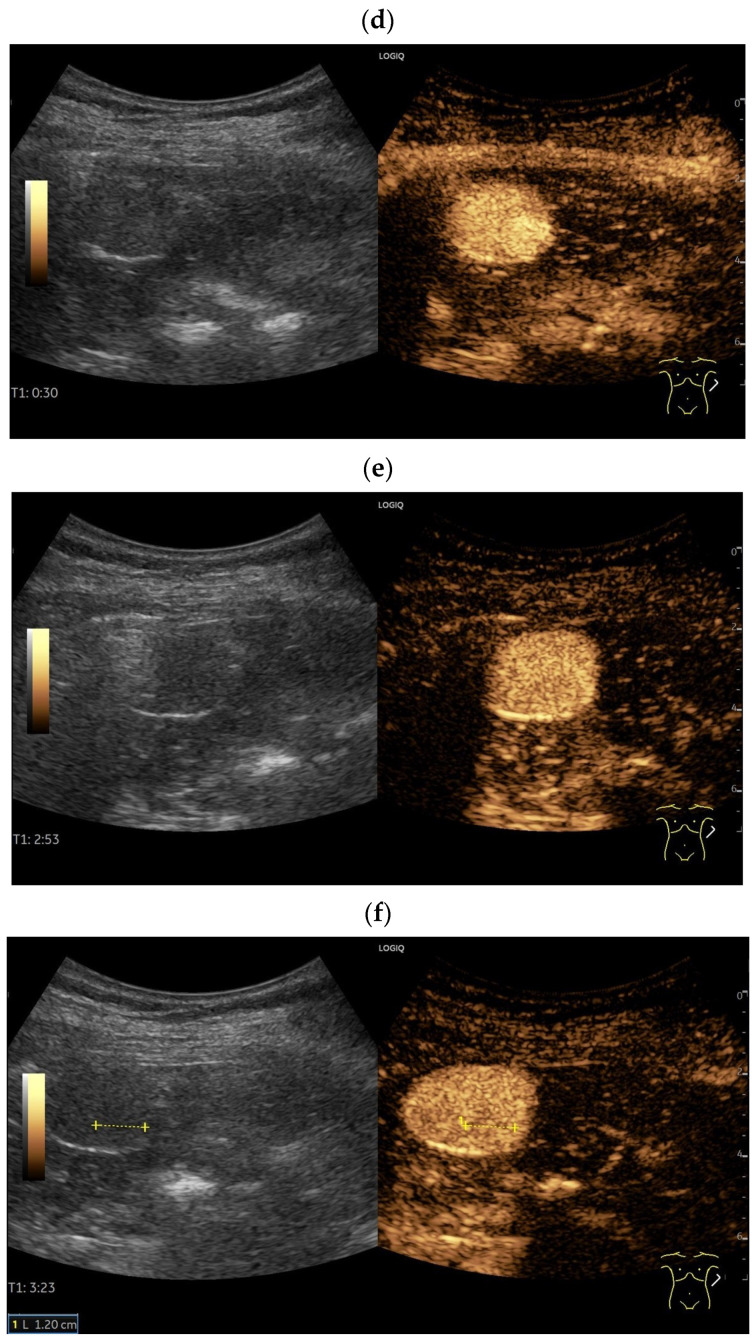
Splenosis with hemangioma. The patient had previously undergone a pancreatic tail resection with splenectomy for a neuroendocrine pancreatic tumor. During follow-up, a 30 mm round, smooth-bordered lesion (yellow arrow) was found in the former splenic bed. This lesion exhibited spleen parenchyma-like echogenicity but also included a 12 mm focal hypoechoic lesion (white arrow). Small vessels were visible on CDI at the periphery of the hypoechoic lesion (**a**). Contrast-enhanced ultrasound (CEUS) using 1.2 mL of SonoVue showed heterogeneous enhancement of the mass in the arterial phase, while the hypoechoic lesion displayed smooth, ring-shaped enhancement (**b**). The enhancement of the mass became homogeneous (**c**). The small lesion showed centripetal enhancement with complete homogeneous hyperenhancement at 30 s (**d**). Subsequently, the small lesion’s enhancement leveled to match that of the surrounding parenchyma at 2:53 min (**e**). Enhancement of the mass remains uniformly intense, while the small lesion showed a faint subtle hypoenhancement (**f**). The finding was classified as splenosis with a small hemangioma. Hyperenhancement was present for over 5 min.

In a study involving 13 patients, peritoneal splenic grafts showed spleen-specific enhancement after SonoVue application [27]. When comparing peritoneal splenoses and peritoneal metastases, the splenoses showed intense enhancement [27]. This was still at 60% of maximum enhancement after 90 s. In contrast, metastases showed varying enhancement with progressive washout. After 90 s, there was a clear distinction between splenoses and metastases. While the enhancement intensity in splenoses remained similar from 40 s to 4 min, it decreased progressively in metastases [27] [Figure 6, Figure 7 and Figure 8].

Similar to the spleen, focal lesions can form within splenoses [Figure 8].

### 4.4. Intrahepatic

Zhong et al. described in their cases, the expected typical CEUS behavior of intrahepatic splenoses with homogeneous hyperenhancement in the arterial phase without washout in the portal or late phases up to 4 min [24]. Nevertheless, differential diagnostic difficulties with some benign liver lesions may arise. Hemangiomas, FNH, and some (inflammatory) hepatocellular adenomas are also hyperenhanced in the late phase on CEUS. Hyperenhancement after 2 min in the noncirrhotic liver is considered a criterion for benign liver lesions [59]. However, all benign liver tumors have a special vascular architecture in the arterial phase and there is no tumor that is enhanced as long-lasting as spleen tissue.

Dölle et al. reported a round to oval configured, hypoechoic, heterogeneous lesion with a hypoechoic rim in the left lobe of a moderately steatotic liver [57]. On CEUS, the lesion showed marginal hyperenhancement in the arterial phase. Contrast filling was centripetal via radial vessels. In the portal venous phase and the late phase, the lesion was hyperenhanced compared to the surrounding liver parenchyma but showed a slight loss of enhancement. Based on contrast behavior, the diagnosis of an inflammatory type of hepatic adenoma (HCA) was favored, although FNH was still another possible diagnosis. An MRI scan also raised the possibility of HCA and HCC within the differential diagnoses. Therefore, an ultrasound-guided biopsy was performed, resulting in the diagnosis of splenic tissue.

Sansone et al. also described the discrepancy between CEUS and MRI in a case of multiple intrahepatic splenoses [58]. The lesions showed persistent hyperenhancement in the portal venous and late phases on CEUS and were suspicious for hemangiomas. On MRI with liver-specific contrast, the lesions were suspicious for adenomas [58]. This case also highlights the diagnostic difficulties of intrahepatic splenoses, which is otherwise easy to identify on CEUS owing to the long hyperenhancement. However, this typical hyperenhancement is not always detectable, as in a case by Cruz et al. [60]. In the late phase, a faint washout of the lesion was visible. The suspected diagnosis was thus an adenoma. Only histological confirmation revealed the intrahepatic splenosis.

**Figure 9:**
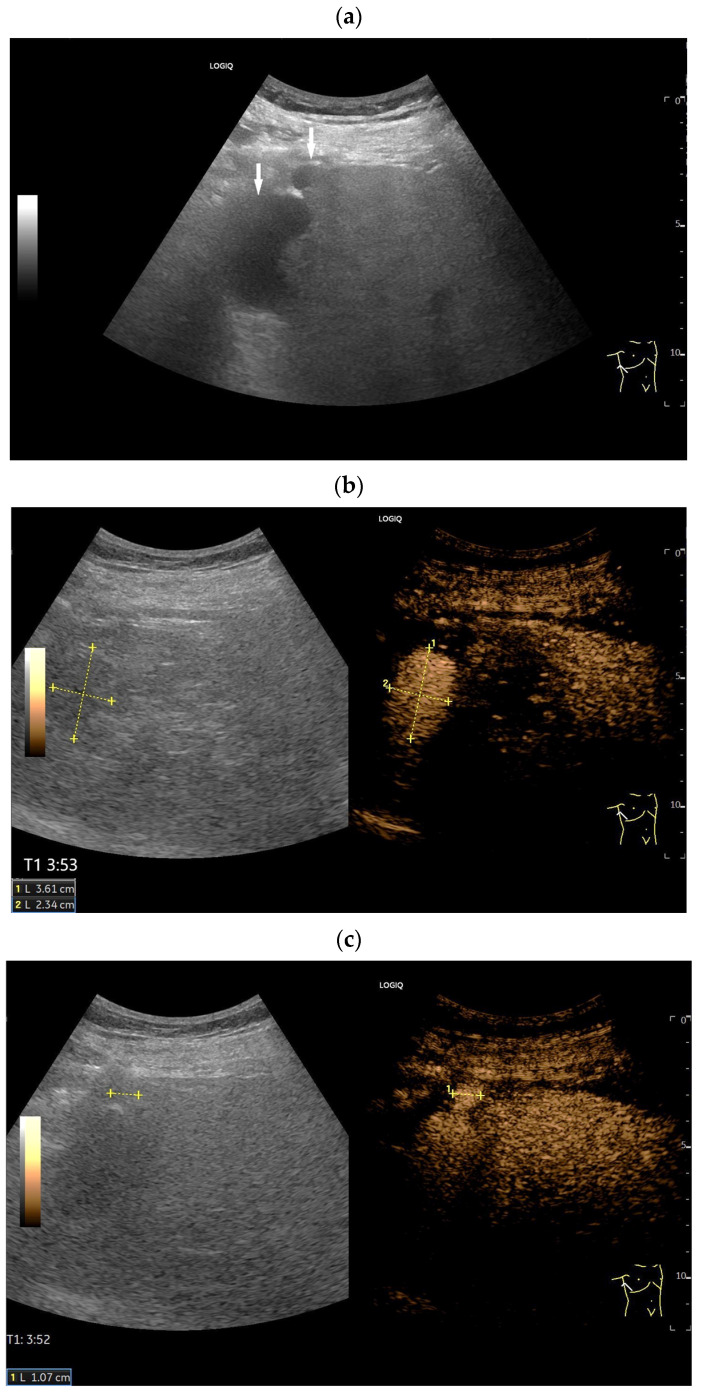

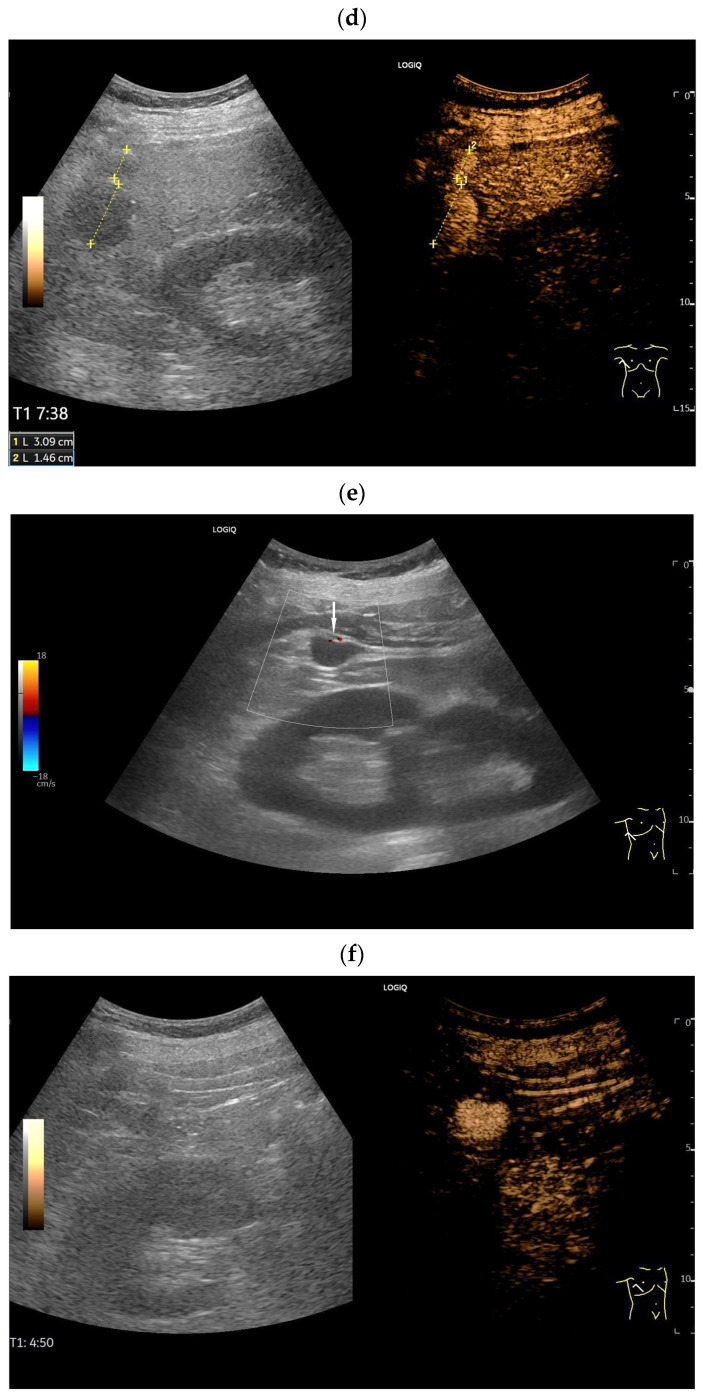

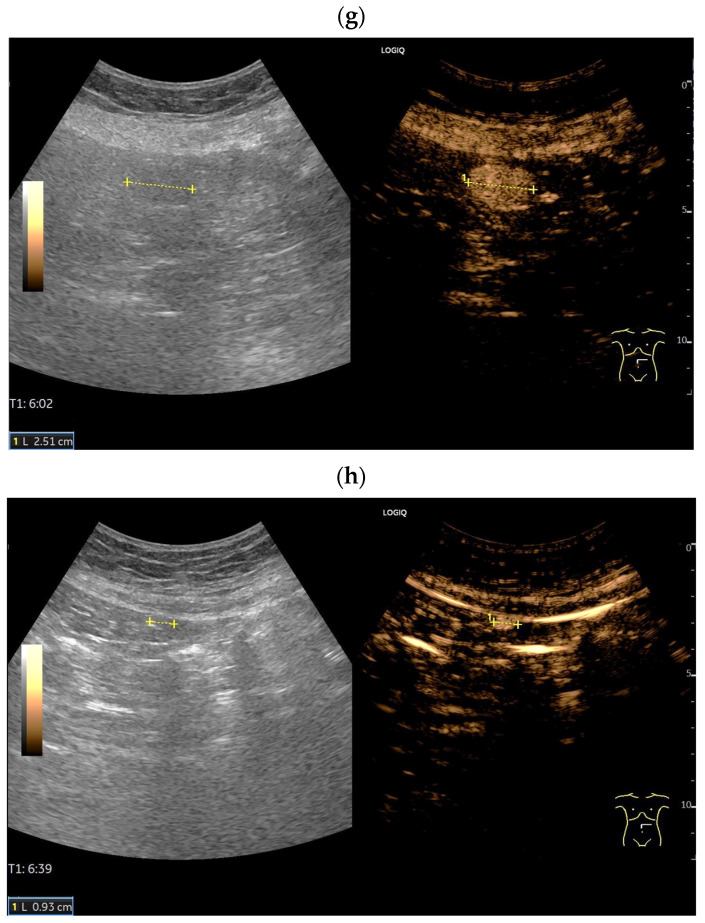
Splenosis-intrahepatic and intraperitoneal. US was performed on a patient with deep vein thrombosis to rule out paraneoplastic syndrome. The patient had undergone a splenectomy more than 10 years ago, following a motorcycle accident. In the liver, two well-defined oval and round hypoechoic lesions (arrows) were observed subcapsular in the right lobe of the liver, with moderate hepatic steatosis (**a**). Multiple round and oval lesions were also visible in the abdomen (arrow) (**b**). Contrast-enhanced ultrasound (CEUS) with 1.2 mL of SonoVue showed homogeneous hyperenhancement of the liver lesions (between the markers), which persisted into the late phase. This was observed at 3:52 min (**c**,**d**) and still at 7:39 min (**e**). The multiple round lesions in the abdomen also showed prolonged enhancement (**f**,**g**), and lesions were revealed that had not been previously visible on just B-mode ultrasound alone (**h**).

In patients at risk for hepatocellular carcinoma (HCC), this is the most important differential diagnosis, but HCC does not show hyperenhancement in the late phase. However, arterially hyperenhanced malignant liver tumors show a washout in the portal venous and late phase or may be isoenhanced like some HCCs in liver cirrhosis and thus can be differentiated from the very prolonged hyperenhancing splenic tissue [Figure 9].

If an oval, smooth-bordered, subcapsular, homogeneous lesion in a patient with a history of splenectomy shows persistent intense hyperenhancement for more than five minutes, then this would be highly suggestive of intrahepatic splenosis. All other liver lesions in the non-cirrhotic liver with hyperenhancement in the late phase are usually benign. There are only a few, such as some of the rare inflammatory hepatocellular adenomas, which require monitoring or, if necessary, therapeutic intervention. Therefore, in cases of doubt, imaging with MRI should always be performed, and if the lesion remains indeterminate, a US-guided biopsy should be considered.

### 4.5. Intrapancreatic Accessory Spleen and Splenosis

Intrapancreatic accessory spleen is rare, and true intrapancreatic splenosis is extremely rare. In most cases, intrapancreatic spleen tissue is hypoechoic to pancreatic parenchyma with a normal pancreatic duct. In patients with no relevant history of abdominal trauma, splenectomy or other abdominal surgery and localization to the tail of the pancreas, an intrapancreatic accessory spleen should be suspected. However, misdiagnosis of IPAS occurs very frequently despite modern imaging modalities and also with fine needle aspiration [61]. To avoid unnecessary surgery, it is important to distinguish intrapancreatic accessory spleen (IPAS) and intrapancreatic splenosis from smoothly bordered, solid, well-vascularized pancreatic tumors. These are predominantly pancreatic neuroendocrine neoplasms (pNEN) and metastases from other tumors, especially renal cell carcinoma. Even rarer hypervascular pancreatic tumors are perivascular epithelioid cell tumor (PEComa) [62] and some mesenchymal tumors [63,64]. Even more challenging is the diagnosis of IPAS from epidermoid cysts and their differentiation from solid-cystic pancreatic tumors [65,66,67]. Here too, the diagnostic value of long-lasting enhancement of splenic tissue over several minutes is evident. In normal pancreatic parenchyma, the peak enhancement is observed at approximately 20–25 s after injection of UCA, before the slow, progressive hypoenhancement of the parenchymal phase starts [68]. Well-vascularized pancreatic tumors, such as neuroendocrine tumors or metastases of renal cell carcinoma, generally show hyperenhancement in the arterial phase, but washout occurs in the parenchymal phase of the pancreas [69]. In contrast to either healthy pancreatic parenchyma or hypervascular pancreatic tumors, the hyperenhancement of splenic tissue is much longer and persists. Roberts et al. demonstrated prolonged hyperenhancement up to 7 min post injection (p.i.) in an intrapancreatic accessory spleen [70].

Xiao et al. described a “slightly high” enhancement in the late phase without specifying the exact time [71].

Kim et al. [72] described 6 cases of intrapancreatic accessory spleens on CEUS with the 1st generation UCA Levovist (Schering AG, Berlin, Germany). In the early arterial phase, all accessory spleens were heterogeneously enhanced but isoechoic to the spleen. In the late arterial phase, 50% were homogeneously enhanced. In the portal venous and late phase, all accessory spleens were homogeneously enhanced. In all phases, enhancement compared with the pancreas was high and similar to the spleen. Interestingly, a vascular hilus could only be delineated in 50% of cases, which is a typical feature described in accessory spleens. The lack of delineation of a vascular hilus does not apparently affect the typical enhancement pattern of splenic tissue [72].

De Robertis et al. [6] describe a case of a splenosis in the pancreatic head. The appearance of the splenosis on CEUS with SonoVue was equal to that of normal splenic tissue: a marked enhancement during the arterial phase and trend to accumulate ultrasound contrast microbubbles in the late phase [6]. The history of a previous splenectomy and the localization of the lesion within the pancreatic head, and not the pancreatic tail, would suggest splenosis rather than an accessory intrapancreatic spleen.

Makino et al. [73] reported a case of an intrapancreatic accessory spleen, in which CEUS using the UCA Sonazoid (Daiichi-Sankyo, Tokyo, Japan) was useful for the diagnosis. Isoenhancement was demonstrated in the postvascular phase compared with the spleen [73].

In contrast to SonoVue, Sonazoid exhibits a post-vascular Kupffer phase and also provides information on the vascularity and uptake within reticuloendothelial system. Similar to SonoVue, Sonazoid is used for the differential diagnosis of liver lesions. We were not able to find any descriptions of the behavior of splenic tissue in CEUS performed with Sonazoid in comparison to SonoVue. There are only single reports on the enhancement of intrapancreatic accessory spleens with SonoVue.

### 4.6. Contrast-Enhanced Low Mechanical Index—EUS (CE-LMI-EUS)

The assessment of the tail of the pancreas and the delineation of smaller pancreatic lesions may be limited in abdominal US. In contrast, endoscopic ultrasound (EUS) has a superior spatial resolution, and access to the pancreatic tail is easy with a very close distance between the transducer and the spleen [74]. Contrast-enhanced low mechanical index EUS (CE-LMI-EUS) has proven to be useful for small pancreatic tumors [75,76], especially for characterizing intrapancreatic ectopic spleen tissue (accessory spleens and post-splenectomy splenosis) [15]. However, there is a lack of large studies documenting the contrast behavior of IPAS (especially in the parenchymal phase) on CE-LMI-EUS with SonoVue.

Marques et al. reported [77] a 5 mm lesion isoechogenic to the spleen in the tail of the pancreas on EUS. This was homogeneously enhanced in the arterial phase on contrast-enhanced LMI-EUS with SonoVue. However, contrast behavior in the parenchymal phase was not described.

Minami et al. [78] described an IPAS with an epidermoid cyst. The solid components showed splenic tissue—similar to enhancement on CE-LMI-EUS with SonoVue. However, no information was provided regarding the duration of the enhancement. In a comparative study by Kano et al. [79] of 4 IPAS and 26 pancreatic neuroendocrine tumors (pNETs) on CE-LMI-EUS with Sonazoid, 75% of IPAS were still hyperenhanced compared to the surrounding pancreatic parenchyma after 60 s, but only 15% of pNETs. After 180 s, 25% of IPAS were hyperenhanced, but not pNET. After 300 s, neither IPAS nor pNET were hyperenhanced, but 100% of IPAS were isoenhanced and 93% of pNET were hypoenhanced [79]. The general expectation that splenic tissue would still be hyperenhanced compared to surrounding pancreatic parenchyma after 300 s was not true using Sonazoid for CELMI-EUS in this study.

Confirmation of an IPAS diagnosis using EUS-guided sampling can be performed with a high success rate. In a large study by Ardengh et al. [80] on 2060 pancreatic EUS-guided samplings, 0.6% were IPAS. Micro histology confirmed the diagnosis of IPAS in 90% (9/10 patients) [80]. Renno et al. [81] report 32 EUS-guided samplings of intrapancreatic spleen tissue. In 25% of the patients, there was a known history of abdominal trauma or surgery. The mass was found in the pancreatic tail in 90.6%, pancreatic head in 6.2%, and body in 3.1%. Echogenicity was hypoechoic at 76.9%, isoechoic in 15.3%, and hypoechoic/isoechoic in 7.6%. The lesion was homogenous in 92.8. Immunostaining for CD8 was positive in 21/32 cases (65.6%), and unreported in the remaining cases. Typical cytologic findings include a heterogeneous population of lymphocytes with traversing small vessels over a background of blood and mixed inflammatory cells. Mixed inflammatory infiltrate, eosinophils, or other splenic elements that resemble red or white pulp may also be seen. CD8 immunohistochemistry delineates the typical pattern of splenic sinusoidal epithelia. Therefore, it is a very specific tool to differentiate accessory spleen from lymphoid tissue [81] and is recommended to diagnose intrapancreatic accessory spleen and splenosis [77,82].

**Figure 10:**
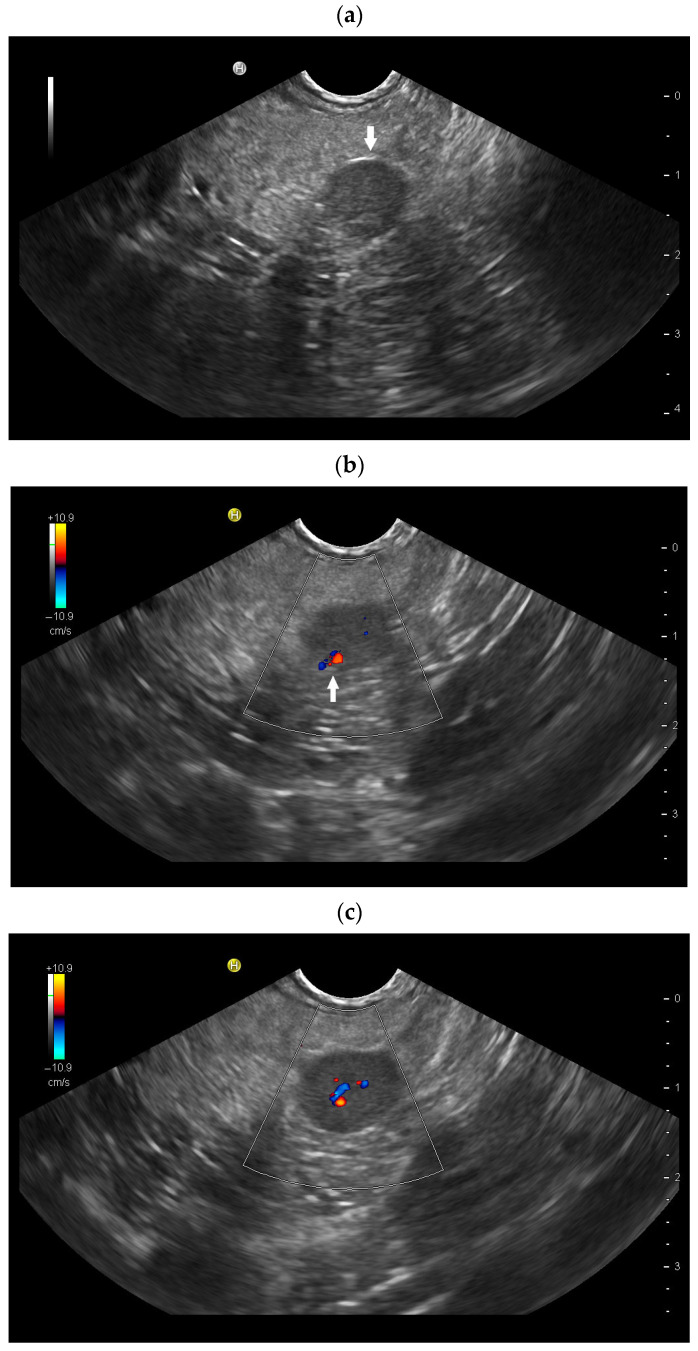

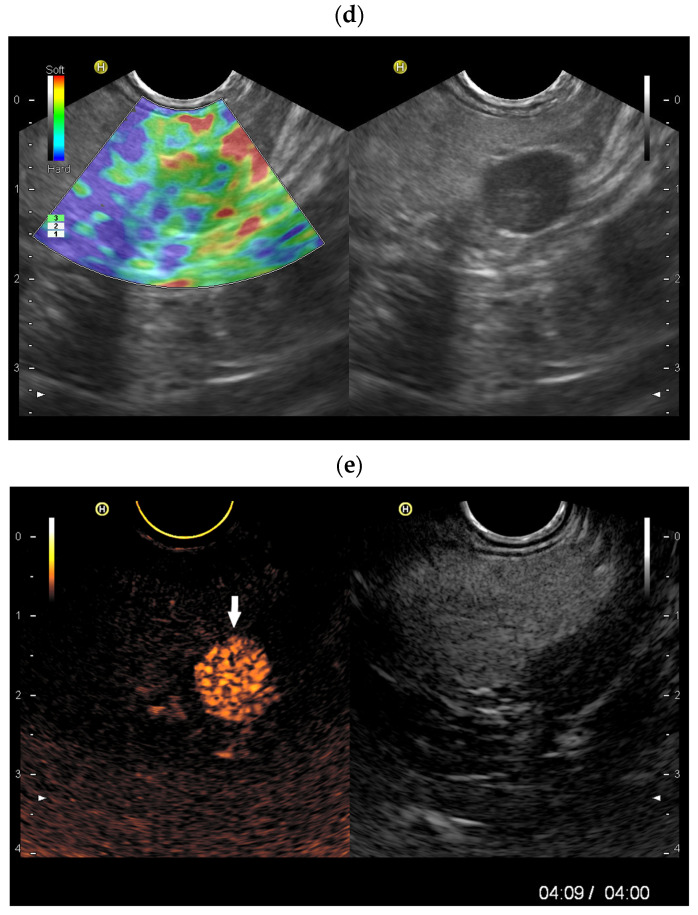
Intrapancreatic accessory spleen. CT scan performed due to abdominal complaints. The CT scan showed a hyperenhanced lesion in the tail of the pancreas. Correspondingly, EUS revealed a smooth-bordered, approximately 11 mm, slightly hypoechoic lesion in the tail of the pancreas (arrow) (**a**). Feeding vessel (arrow) (**b**) and central vessels (**c**) were visible. Elastography showed that the lesion was not stiffer than the surrounding pancreatic tissue (**d**). CE-LMI-EUS (SonoVue) demonstrated a hyperenhanced lesion. The examination was terminated after four minutes; the lesion (arrow) remained intensively hyperenhanced (**e**). These findings are typical of an intrapancreatic accessory spleen. EUS-FNA was thus not performed. Further investigations and follow-up were considered unnecessary owing to the highly specific findings.

**Figure 11:**
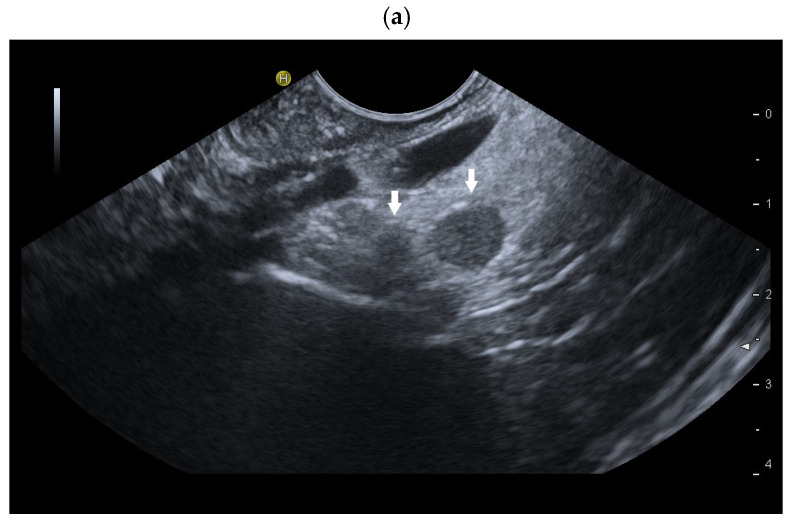

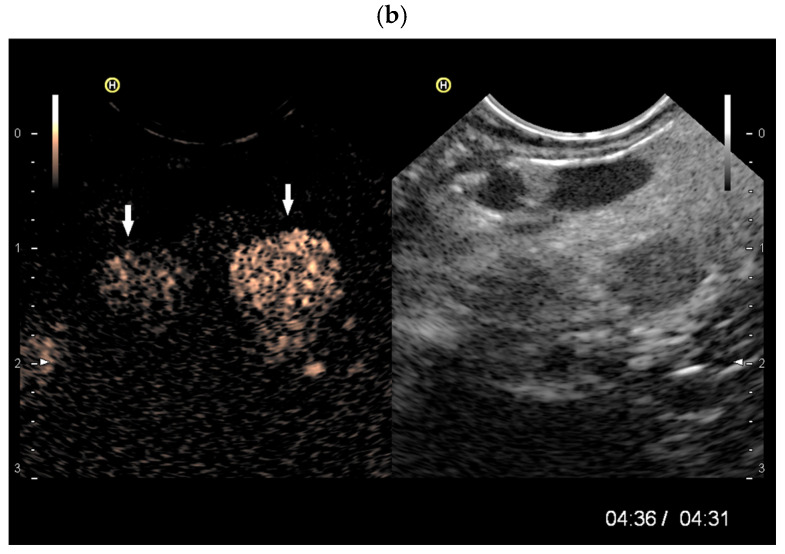
Intrapancreatic accessory spleens. EUS was performed to rule out choledocholithiasis. Incidental finding of a 9 mm, smooth-bordered, slightly hypoechoic lesion at the tail of the pancreas. A second, smaller hypoechoic lesion was only faintly visible on B-mode EUS (arrows) (**a**). In contrast-enhanced LMI-EUS (CE-LMI-EUS), the intrapancreatic lesion remained hyperenhanced for several minutes. Surprisingly, a second, identically hyperenhanced lesion was discovered. The examination was performed for up to 4:30 min, and enhancement of the lesion was still visible (**b**). The diagnosis of two small intrapancreatic accessory spleens was established. EUS-FNA, further diagnostic measures or follow-up examinations were not necessary.

In our experience, enhancement of the pancreatic parenchyma and of well-vascularized tumors in the parenchymal phase is very limited in CE-LMI-EUS using SonoVue. If a smoothly bordered, homogeneous, hypoechoic pancreatic lesion without pancreatic duct dilatation still shows enhancement after more than 4 min, we would classify this as IPAS [Figure 10 and Figure 11]. However, one should adhere to the study data, which described hyperenhancement after 5 min for splenic tissue [2]. In all indeterminate cases, EUS-guided sampling must be performed. The pathologist should be informed of the possible diagnosis of IPAS so that targeted examinations can be carried out.

There are numerous case reports of splenosis and accessory spleens with descriptions of diagnostics using CT, MRI, targeted scintigraphy and their outcomes. However, descriptions using CEUS are rare. In Table 4, we have compiled all descriptions of splenosis, IPAS and splenovisceral fusion using CEUS.

### 4.7. Critical Analysis

According to the reported studies, the long-lasting contrast enhancement of splenic tissue for up to 5 min or more is described as typical for the spleen. The EFSUMB guideline also advocates CEUS as a suitable method for diagnosing ectopic spleen tissue. Unfortunately, however, there are only studies describing clinical cases of ectopic and heterotopic spleen with CEUS. With the exception of one comparative study [27], there are no further prospective studies or pooled analysis of diagnostic accuracy.

In the case reports, the description of the vascular phases and the timing of the enhancement are inconsistent. The duration varies between studies (in most studies: 4–7 min). Precise description of enhancement timing is also missing in some reports. Moreover, the terminology used to describe the enhancement is also not consistent between studies, leaving room for interpretation. No estimates of sensitivity/specificity, interobserver variability, or statistical validation can be derived from the case reports researched. Since clinical practice mainly involves isolated case observations, future prospective and multicenter studies would be desirable to better define the diagnostic performance and reproducibility of CEUS in ectopic or heterotopic splenic tissue. Unfortunately, it is not possible to make a general recommendation that CEUS is used as the sole method for diagnosing ectopic spleen tissue. However, if ectopic splenic tissue is clinically suspected, CEUS should be performed and the examination extended intermittently to at least 5 min. The extent of enhancement should be assessed after 5 min or later.

### 4.8. Ectopic and Heterotopic Spleen Tissue on CT, MRI and Scintigraphy

CT findings of splenosis are also nonspecific—discrete hypodensity on non-contrast-enhanced images, with homogenous enhancement on delayed arterial phase and slight enhancement on venous phase [22].

On a T1-weighted MRI image, the splenosis reveals itself as a homogeneous signal-weak structure with a thin hypointense border representing the fibrous capsule, which also shows hypointensity on a T2-weighted image (T2WI). On a T2WI, it appears as a homogeneous isointense to hyperintense structure, just like normal spleen tissue, and can be easily compared to a normal spleen [22].

Scintigraphy studies include Tc99m heat-damaged autologous red blood cell (RBC) scintigraphy and Tc-99m sulfur colloid scintigraphy. Tc99m RBC imaging is more specific, as 90% of Tc-99m-labeled autologous RBCs are taken by reticuloendothelial cells, which is helpful in identifying spleen tissue [22]. For further details on the appearance of spleen tissue under CT, MRI, and spleen-specific scintigraphy, please refer to the related specialized literature.

## 5. Conclusions

**Figure 12:**
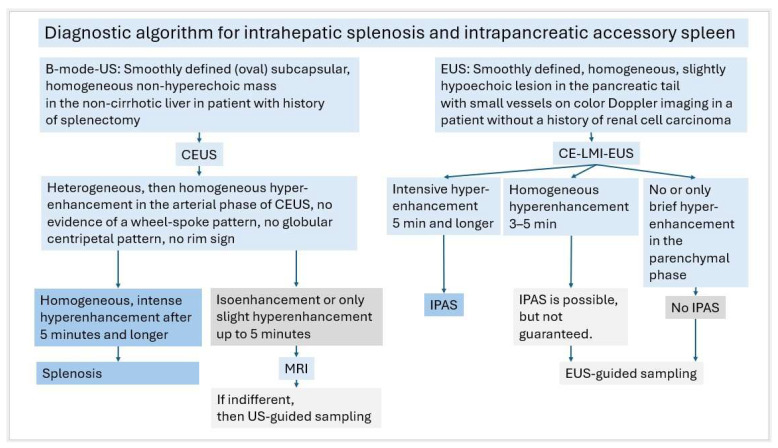
Diagnostic algorithm for intrahepatic splenosis and intrapancreatic accessory spleen (IPAS).

On CEUS with SonoVue, splenic tissue exhibits the unique characteristic of long-lasting enhancement past 5 min, which can be used to diagnose heterotopic and ectopic spleen tissue. It is complementary to other imaging modalities and can help avoid unnecessary intervention [Figure 12].

## 6. Take-Home Message

- Well-defined, well-vascularized lesions in the left abdomen may represent accessory spleens or splenomas, and must be distinguished from well-vascularized tumors.
- Contrast-enhanced ultrasound (CEUS) should be routinely used for the differential diagnosis of these lesions, particularly following splenectomy or abdominal trauma.
- Prolonged contrast enhancement lasting ≥ 5 min is considered a strong indication of splenic tissue (e.g., accessory spleen or splenosis).
- In the non-cirrhotic liver, only benign lesions show a late hyperenhancement phase; however, their intensity is usually lower than that of splenic tissue.
- Peritoneal splenoses can often be clearly distinguished from peritoneal metastases using CEUS, so that further diagnostic testing is frequently unnecessary.
- An intrapancreatic accessory spleen is usually found in the pancreatic tail; in cases of uncertainty, CE-EUS and, if necessary, targeted biopsy are indicated.
- Congenital splenovisceral fusions (e.g., splenogonadal or splenorenal) can mimic tumors and should be considered if corresponding findings are present.
- Multimodal imaging and targeted sampling are advisable in cases of diagnostic uncertainty to avoid unnecessary surgical resections.

## Figures and Tables

**Table 1:** Heterotopic and ectopic spleen tissue.

Classification	Explanation/Cause/Differentiation
**Heterotopic spleen tissue**	
Accessory spleen	Small spherical extra spleens in or near the hilum of the spleen, in the left abdomen, in the tail of the pancreas and rarely in other locations (e.g., stomach, gonads, adrenal glands, and gonads) as a result of incomplete fusion of the spleen tissue in its embryogenesis.
Splenovisceral fusion	Congenital fusion of spleen tissue with organs, inclusion of spleen tissue in organs. Not a result of autotransplantation following spleen injury or splenectomy.
-Splenogonadal fusion	Most important splenovisceral fusion.
Discontinuous fusion	A connecting fibrous band between the left gonad and the spleen is present or absent.
Continuous fusion	
-Splenorenal, splenoadrenal	Congenital, without mechanisms of autotransplantation.
-Splenopancreatic	or intrapancreatic accessory spleen (IPAS), or pancreatic tissue in spleen tissue, or fusion of the pancreatic tail with the splenic hilum.
-Splenohepatic	Congenital, without mechanisms of autotransplantation.
**Ectopic spleen tissue**	
Splenosis	Autotransplantation of spleen tissue after splenic injury and/or splenectomy.
“Wandering spleen”	Dystopic spleen due to weakness or maldevelopment of splenic ligaments.
Polysplenia	Within the context of heterotaxy syndromes.
Asplenia	

**Table 2:** Difference between accessory spleen and splenosis.

	Accessory Spleen	Splenosis
Cause	Congenital	Due to autotransplantation of spleen tissue after trauma, splenic injury and/or splenectomy.
Localization	Usually in the splenic hilum	Distributed throughout the abdomen, peritoneum, or embedded as spherical masses in organs.
Capsule	Strong capsule present	Weak/thin capsule or pseudocapsule.
Echogenicity	Echogenicity and tissue pattern on ultrasound identical to the spleen	A direct comparison of the spleen is difficult, as the spleen is usually missing as a result of splenectomy.
Vascular supply	From branches of the splenic artery	Supply from surrounding tissue.
Histology	Identical to spleen tissue	Imperfect white pulp in some cases.

**Table 3:** Differential diagnostic significance of accessory spleen, splenosis, and splenovisceral fusion.

Splenic Condition	Important Differential Diagnoses
Accessory spleen	Large accessory spleens in the hilum of the spleen can mimic pancreatic tail tumors, and multiple accessory spleens can mimic pathological lymph nodes.
Intrapancreatic accessory spleen and splenosis	Well-vascularized, smoothly bordered hypoechoic tumors that do not lead to obstruction of the pancreatic duct: neuroendocrine tumors, metastases from renal cell carcinomas.
Intrahepatic splenosis in liver diseases	Hepatocellular carcinoma, hemangioma.
Intrahepatic splenosis without liver diseases	Hemangioma, hepatocellular adenoma, focal nodular hyperplasia.
Peritoneal splenosis	Metastases, lymphomas.
Pelvic splenosis	Ovarian tumors, lymphomas.
Gastrointestinal splenosis	GIST, intestinal tumors.
Intrathoracic splenosis	Mesothelioma, lymphoma, thymoma, other solid tumors.
Splenogonadal fusion	Scrotal tumor.
Splenorenal fusion	Renal cell carcinoma, other solid renal tumors.

**Table 4:** Enhancement characteristics of histologically confirmed splenosis, accessory spleen, and splenovisceral fusion on CEUS. Explanation: p.i.: post-injection. “n.i.” stands for “no information”.

Study	Organ Number of Patients	Splenectomy Yes/No	Arterial Phase	Portal Venous/Venous Phase	Late Phase	Comments
				Parenchymal Phase in Nonhepatic Lesions		
Ota 2005 [83]	Peritoneal midline (n = 1)	Yes	n.i.	Strong enhancement after 6 min		UCA Levovist was used
	Peritoneal right flank (n = 1)			Moderate enhancement after 4 min		
Bertolotto 2009 [27]	Peritoneal (n = 13)	Yes	Intense hyperenhancement	40–240 s p.i. consistent hyperenhancement of 60% of the maximum enhancement.		
Foh 2022 [28]	Multiple abdominal masses	Spleen “was missing”	n.i.	Hyperenhancement (No exact time specified)		Untreated hepatitis C
Ferraioli 2006 [84]	Liver segment VII (n = 1)	yes	Hypoechoic to the surrounding liver	Hyperechoic	Hyperechoic (60–240 s)	Treated from hepatitis C
Sansone 2020 [58]	Liver (n = multiple)	yes	n.i.	Hyperenhancement	Persistent Hyperenhancement	(No exact time specified)
Dölle 2021 [57]	Liver segment II (n = 1)		Hyperenhancement, centripetal filling and a spoke wheel pattern	Hyperenhancement	Hyperenhancement; with low central enhancement loss	Image documentation up to 2:11 min
Zhong 2021 [24]	Liver (left lobe n = 2)	yes	Homogeneous hyperenhancement	Persistent hyperenhancement	Persistent hyperenhancement	Examination lasting 5 min
	Liver (right lobe n = 1)		Homogeneous hyperenhancement	Persistent hyperenhancement	Persistent hyperenhancement	
	Nodule in the gastrohepatic ligament (n = 1)		n.i.	Persistent hyperenhancement		
Liu 2022 [85]	Liver (left lobe) (n = 1)	yes	High enhancement	Slightly high enhancement	Slightly high enhancement	(No exact time specified)
Chen 2023 [86]	Liver (n = 7)	6/7 yes	Hyperenhancement Fast complete n = 5/7 Centripetal n = 2/7	Hyperenhancement n = 2/7 Isoenhancement n = 5/7	Hyperenhancement or isoenhancement n = 7/7	(No exact time specified)
Möller 2023 [87]	Right liver lobe (n = 1), two subcapsular lesions	yes	Heterogeneous and less enhanced than surrounding liver	Homogeneous hyperenhancement	Homogeneous hyperenhancement, documentation up to 6:10 min	
Xiao 2024 [71]	Liver segment II (n = 1)	yes	High enhancement	high enhancement	high enhancement (at 5 min)	
	Liver segment II (n = 1)	yes	Hyperenhancement	Isoenhancement	Isoenhancement (at 5 min)	chronic hepatitis B cirrhosis
Cruz 2024 [60]	Right liver lobe, subcapsular (n = 1)	yes	rapid centripetal enhancement, hyperenhancement	remaining enhanced	faint washout at 3:31 min	
Ota 2004 [88]	Pancreatic tail (n = 1)	no	n.i.	Enhancement identical to the spleen 5 min after injection		UCA Levovist was used
Kim 2007 [72]	Pancreatic tail (n = 6)	no	Hyperenhancement	Hyperenhancement (3–5 min).		UCA Levovist
Rogers 2010 [70]	Pancreatic tail	no	Inhomogeneous enhancement	Intense strongly enhancement at 7 min p.i.		
Makino 2011 [73]	Pancreatic tail			Similar enhancement in the vascular and postvascular phases		UCA Sonazoid
De Robertis 2014 [6]	Pancreatic head (n = 1)	yes	Homogeneous enhancement	Homogeneous enhancement		(No exact time specified)
Marques 2016 [77]	Pancreatic tail (n = 1)	Yes	hyperenhanced	n.i.		EUS, CE-LMI-EUS
Tatekawa 2018 [89]	Pancreatic tail (n = 1)	Yes	Hyperenhancement	n.i.		UCA Sonazoid
Luo 2020 [90]	Pancreatic tail (n = 1)	No	“Low enhancement”	n.i.		IPAS with squamous epithelial cyst in pancreas
Xiao 2024 [71]	Pancreatic tail (n = 1)	no	hyperenhancement	Hyperenhancement (at 5 min)		
Kruger, Freeman 2018 [29]	Pelvic mass	Motorcycle accident with spleen scars	Heterogeneous enhancement	Hyperenhancement lasting more than 7 min p.i.		A number of small enhancing peritoneal nodules
Foh 2022 [28]	Pleural masses (multiple, n = 2 on CEUS)	Spleen was missing	n.i.	Hyperenhancement (Image documentation up to 4 min)		
Kroenig 2022 [39]	Pleural mass (n = 1)	yes	Strong enhancement	Strong enhancement (in the parenchymal phase), image documentation up to 6 min)		
Trottmann 2015 [91]	Testis (splenogonadal fusion) (n = 1)	No	Homogeneous hyperenhancement	Homogeneous hyperenhancement		(No exact time specified)
Grosu 2019 [46]	Testis (Splenogonadal fusion) (n = 1)	No	Early strong hyperenhancement, homogeneous hyperenhancement	Hyperenhancement		(No exact time specified)

## Data Availability

Not applicable.
